# Comparison of Composite Materials Designed to Optimize Heterogeneous Decatungstate Oxidative Photocatalysis

**DOI:** 10.3390/molecules30173597

**Published:** 2025-09-03

**Authors:** Julia Ong, Benjamin Cajka, Juan C. Scaiano

**Affiliations:** Department of Chemistry and Biomolecular Sciences, University of Ottawa, Ottawa, ON K1N 6N5, Canada; jong017@uottawa.ca (J.O.); bcajk083@uottawa.ca (B.C.)

**Keywords:** decatungstate, photocatalysis, triplet state, alcohol oxidation, ultraviolet irradiation, supported catalysts, green chemistry

## Abstract

Catalysis plays a pivotal role in green chemistry practices, particularly in reducing waste generated during chemical synthesis. Decatungstate (DT) emerges as a potent photocatalyst for Type I oxidations, exhibiting remarkable resilience to oxygen quenching, a characteristic that sets it apart from other excited triplet state photocatalysts. While homogeneous DT catalysis demonstrates effectiveness, its solubility poses challenges for its separation and recycling. To address these limitations, we focus on the development and comparison of heterogeneous DT photocatalysts, aiming to optimize their yield, recovery, and reusability. We synthesized tetrabutylammonium decatungstate (TBADT)-supported catalysts using silica, alumina, titanium dioxide, and glass wool and characterized them using diffuse reflectance measurements. Subsequently, we evaluated their photocatalytic performance by monitoring the oxidation of 1-phenylethanol and cyclohexanol under UVA irradiation. Our findings reveal that TBADT@silica emerges as the most effective catalyst, achieving approximately 20% conversion of cyclohexanol and 50% conversion of 1-phenylethanol with good reusability. Interestingly, we observed that 3-aminopropyl-triethoxysilane (APTES) treatment, intended to enhance DT anchoring, unexpectedly quenches the ^3^DT* triplet state, reducing catalytic activity. This unexpected finding underscores the importance of careful consideration in designing robust and recyclable heterogeneous decatungstate catalysts. Our research contributes significantly to the advancement of heterogeneous photocatalysis, paving the way for future applications in flow photochemistry. Further, we share a Python code (Google 3.12.11) to correct spectra obtained in Cary spectrometers.

## 1. Introduction

Polyoxometalates (POMs) are a diverse class of inorganic metal–oxygen clusters that have garnered significant attention as catalysts for various chemical transformations. Among them, the decatungstate anion, [W_10_O_32_]^4−^ (commonly abbreviated as DT), stands out due to its unique photochemical properties and high reactivity, particularly in its photoexcited state [1,2,3,4,5,6]. Under ultraviolet or near-ultraviolet irradiation, DT transforms into a potent oxidizing agent, capable of abstracting hydrogen atoms from organic substrates or participating in electron transfer processes. Notably, DT participates only in Type I oxidations, which involve radicals or radical ions, but does not involve singlet oxygen chemistry [7].

While homogeneous decatungstate catalysis has demonstrated effectiveness in various reactions, such as alcohol oxidation, sulfide oxidation, and C-H functionalization, its solubility in conventional reaction media poses challenges for catalyst separation and recycling. Consequently, extensive research has focused on the heterogenization of DT, aiming to leverage its inherent catalytic advantages while incorporating the practical benefits of solid catalysts, including ease of recovery, reuse, and suitability for flow reactor systems [8,9,10,11,12,13].

While significant progress has been made in immobilizing decatungstate onto various supports and within different matrices, resulting in recyclable catalysts for crucial chemical transformations, there is still ample room for improvement. Enhancing these materials is crucial for achieving effective oxidations, catalyst recoverability, and reusability, ultimately paving the way for flow photocatalysis. A few studies have incorporated DT onto silica, alumina, carbon dots, and organic resins, but they have not compared the detailed performance of various materials under standardized illumination conditions [8,9,12,14]. Additionally, other studies have incorporated various dopants, such as transition metals; however, while these are very interesting, this last group will not be the focus of this contribution [15,16].

Decatungstate photooxidations have traditionally been mediated by an intermediate labeled as “wO,” which can readily transfer hydrogen atoms (HAT) and exhibits properties reminiscent of triplet states, particularly carbonyl triplets, such as that from benzophenone [17]. However, there was hesitation to identify “wO” with the DT triplet because triplets are easily quenched by oxygen, while “wO” is not. In a recent study, we characterized the DT triplet using time-resolved NIR luminescence [18]. Our study revealed that the DT triplet state energy is only 21 kcal/mol, which is less than the minimum of 23 kcal/mol required to sensitize singlet oxygen [17]. This finding allowed us to identify “wO” as the triplet state of DT, which we labeled as “^3^DT*”.

In our research, we have investigated various supports, such as silica, alumina, glass wool, and titanium dioxide, with decatungstate (DT) as the functional group. DT typically has either sodium (NaDT) or tetrabutylammonium (TBADT) as counterions. In some cases, we also derivatized the supports with 3-aminopropyl-triethoxysilane (APTES) to explore its influence on DT immobilization. Our primary focus is on mechanistic aspects, so we did not delve into the full scope of the reaction. Instead, we chose one aromatic alcohol, 1-phenylethanol, and one aliphatic alcohol, cyclohexanol, as substrates. Both alcohols oxidize to their corresponding ketones, enabling us to examine how the supports and excitation wavelengths impact the reaction outcome. In a recent study, we investigated the role of hydroperoxides in the DT-mediated homogeneous photooxidation of 1-phenylethanol and 1,4-cyclohexadiene. Some of the analytical methods employed in this report are also utilized in our current study.

## 2. Results and Discussion

We initially present a report on the synthesis and characterization of the nanostructures later used as catalysts. More details are presented in the Supplementary Information.

### 2.1. Preparation and Characterization of Materials

#### 2.1.1. Catalyst Preparation

**Figure 1:**
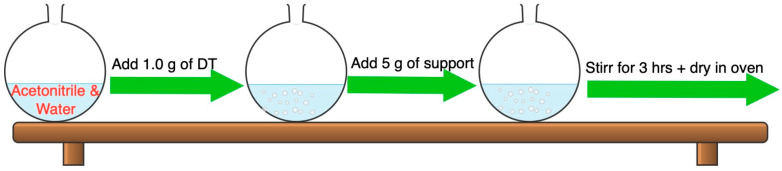
Procedure for the synthesis of heterogeneous decatungstate supported on silica, alumina, TiO_2_, or glass wool.

In this study, silica (SiO_2_), alumina (Al_2_O_3_), titanium dioxide (TiO_2_), and glass wool were chosen as supports for the preparation of heterogeneous catalysts. The synthesis process involved three main steps. Initially, 1 g of decatungstate salt was dissolved in a mixture of 60 mL acetonitrile and 15 mL deionized water in a spherical flask equipped with a rotary evaporator. Subsequently, 5 g of the selected support material was added to the solution. The resulting suspension was subjected to rotation for 3 h, followed by evaporation over 2 h at 30 °C. Finally, the solid was dried overnight in an oven at 120 °C. The procedure is graphically summarized in Figure 1, leading to the catalytic materials used in this contribution.

In some cases, the support was treated with APTES, and in the case of silica, a commercial silica pre-treated with APTES was used. This modification was tested in the hope that APTES would enhance the anchoring of DT. However, in general, these materials did not show any improvement over the untreated material, as APTES proved to be an effective quencher for ^3^DT*. Such quenching was verified using time-resolved NIR spectroscopy and limits the opportunity for triplet DT to react with the substrates of interest (see Supplementary Information for details).

#### 2.1.2. Catalyst Diffuse Reflectance and BET Characterization

**Figure 2:**
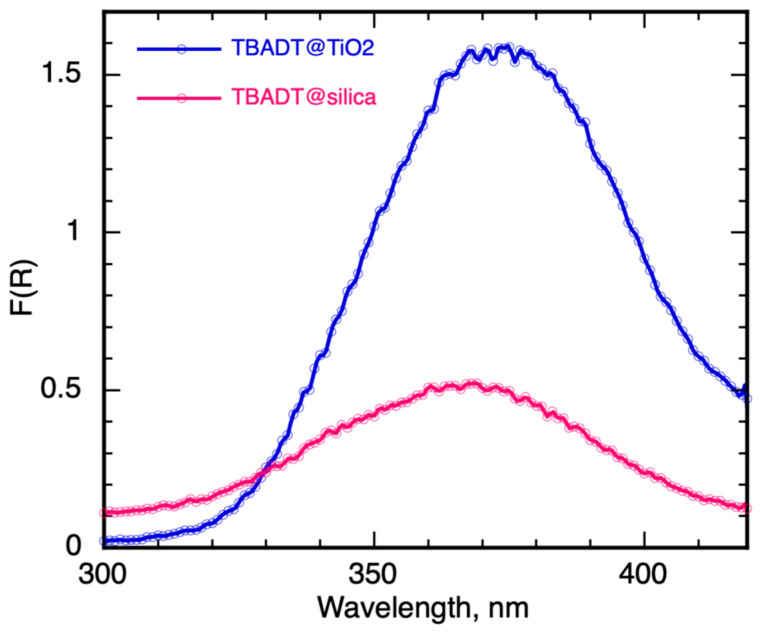
DR spectra for TBADT@TiO_2_ (blue) and TBADT@silica (red) recorded using the respective support as a reference.

Diffuse reflectance (DR) measurements were conducted on heterogeneous catalysts, including glass wool, silica, alumina, and titanium dioxide, to assess the successful attachment of DT to the supports. These measurements are typically performed against a totally reflective standard, but in our case, we opted to use Spectralon as the reference, except for DT@TiO_2_, where powdered TiO_2_ was the reference. This ensured that the recorded spectra clearly indicated whether DT had been adsorbed and if its absorbance was suitable for the UVA irradiation employed in most of the experiments. Figure 2 shows representative DR spectra, while additional data are provided in the Supplementary Information. As already noted, the spectra in Figure 2 have been recorded with the corresponding support, TiO_2_ or spectralon, as a reference. All other DR spectra are included in the Supplementary Information. Even for F(R) ~ 0.5, about 50% of the light is reflected, based on the Kubelka–Munk expression [19]. Both maxima are red-shifted with respect to solution values. The larger shift for TBADT@TiO_2_ likely reflects that, with a band gap of ~3.1 eV, the TiO_2_ reference essentially absorbs all the light in the shorter region of the UVA spectrum.

The data in Figure 2 confirms that the DT absorbance in these materials is adequate for the UVA light sources employed (see Supplementary Information).

Surface BET studies are included in Table S4 and show TBADT@silica as the material with the largest surface at 102.5 m^2^/g.

### 2.2. 1-Phenylethanol Sample Irradiation Using TBADT on Various Supports

**Figure 3:**
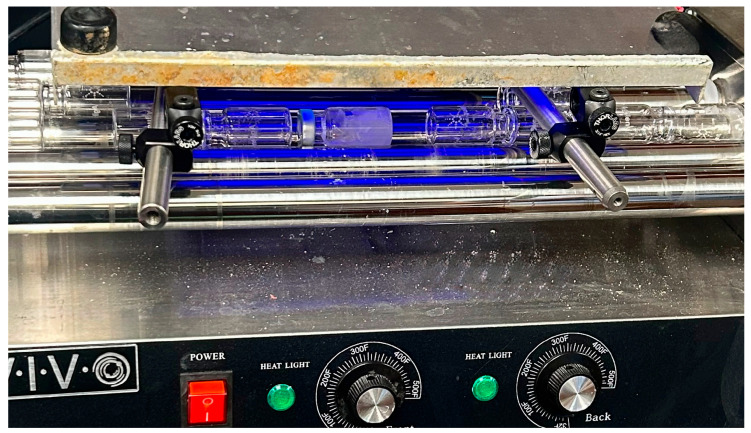
Samples are rotated in a hot-dog cooker (heating off) while illuminated with appropriate light sources, UVA in this image. Empty vials are usually added to stop the samples from drifting from the preferred position under the light source.

Preliminary experiments were performed in 10 mL fused silica tubes (Luzchem Q-tubes) with 7 mL of liquid and connected to an oxygen-filled balloon, as in previous work [6]. However, we found that the simpler approach described below was easier, highly reproducible, and therefore adopted for all the work in this contribution. This approach is illustrated in Figure 3. Samples were sealed with a crimp tool and Teflon-coated caps in 10 mL quartz vials. Each vial contained 5 mL of the sample suspension, and the sealed atmosphere was pure oxygen. To ensure continuous, gentle stirring, the samples were rotated at room temperature using a commercial hot dog cooker. To evaluate the reusability of the catalyst, the reaction mixture was centrifuged at 5000 rpm for 5 min to separate the solid catalyst from the solution. The supernatant was carefully removed. The recovered solid catalyst was then washed thoroughly with hexane (4 × 10 mL) to remove any residual organic compounds or reaction byproducts. Each washing step involved vortexing followed by centrifugation. After the final wash, the catalyst was dried in an oven at 120 °C for 24 h. This recycling procedure was repeated twice to assess catalyst stability and potential loss of activity.

Additionally, the samples were illuminated from the top using UVA light (from Luzchem LES-UVA lamps, Luzchem Research (Ottawa, ON, Canada)) or 280 nm light from a Violumas 12 LED strip. The emission spectra of these light sources are provided in the Supplementary Information. The DT composites function as their own sensors, ensuring that oxygen is not depleted. We frequently observe the characteristic blue color of DTH_2_ when irradiation is stopped. If the color fades within a few minutes, it is a reliable indicator that oxygen is still present.

**Figure 4:**
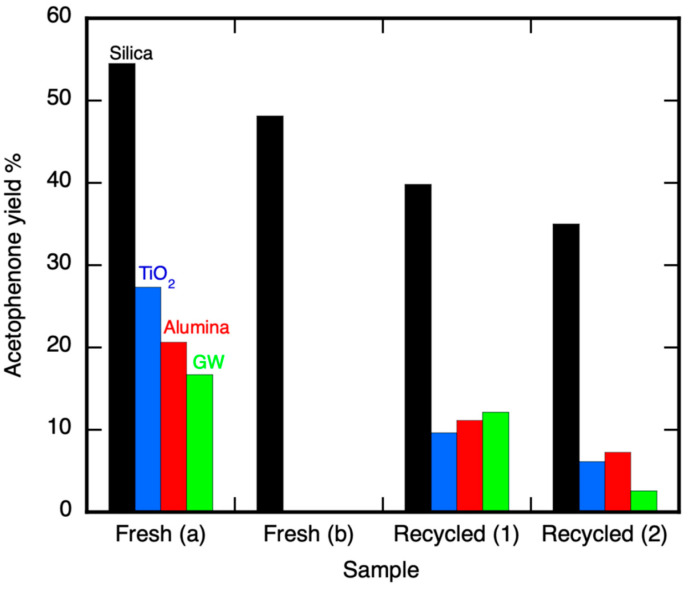
UVA irradiation of 1-phenylethanol (0.1 M) in chloroform for 24 h under oxygen was performed using silica (black), TiO_2_ (blue), alumina (red), or glass wool (green) as supports for TBADT. The supports were not pre-treated with APTES. The silica sample was run in duplicate, and all samples were recycled twice. The details of the sample preparation for recycling are provided in the Materials and Methods section.

In Figure 4, we present the results that will serve as the foundation for our discussion. In the setup depicted in Figure 3, all samples of 1-phenylethanol were subjected to UVA radiation for a duration of 24 h. Subsequently, a gas chromatography–mass spectrometry (GC-MS) analysis was performed to ascertain the conversion of 1-phenylethanol to acetophenone. The reactions exhibited remarkable cleanliness, with only occasional detection of trace amounts of toluene or styrene.

**Scheme 1:**
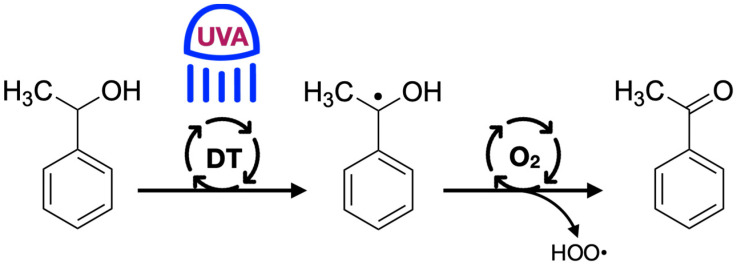
Oxidation of 1-phenylethanol in the presence of UVA light and DT photocatalysts.

Figure 4 illustrates that yields of approximately 50% over 24 h are achieved using fresh samples of the TBADT@silica system. The remaining 50% is largely unreacted 1-phenylethanol, and the yields decrease by 5–10% for subsequent photocatalytic cycles. While all other supports exhibit comparable results, the yields are significantly lower. TBADT@silica recycles rather well at least 2 times, but an attempt to recycle a third time led to yield of only 5%, largely due to the loss of catalytic material (60 mg of the initial 140 mg). Notably, the lowest yield of about 17% was obtained for TBADT@GW. Importantly, this material is the most easily adaptable for flow photocatalysis, and it may be worthwhile to revisit and optimize it in future studies. Scheme 1 illustrates the oxidative pathway for the DT photocatalyzed conversion of 1-phenylethanol into acetophenone [6]; a similar reaction path also applies to the oxidation of cyclohexanol.

**Figure 5:**
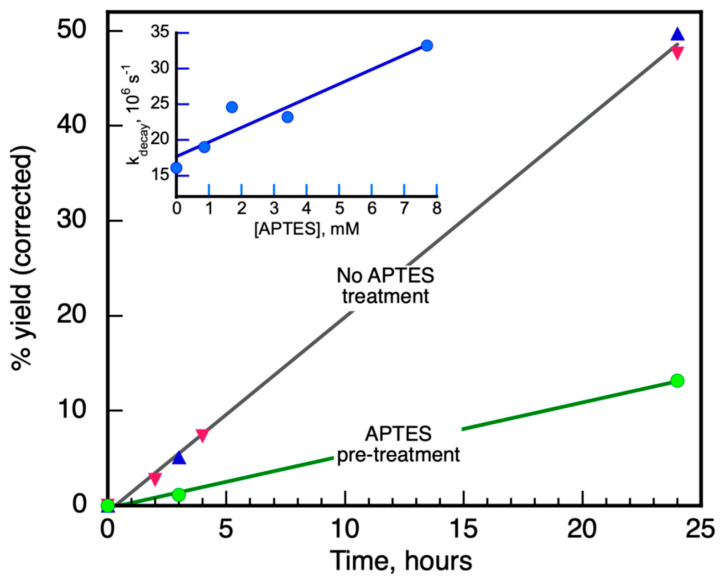
The main plot shows the evolution of the acetophenone yield with time for irradiations in the setup shown in Figure 3. The lines show (red and blue triangles) a linear fit in both cases, with correlation coefficients > 0.999. The red and blue triangles correspond to duplicate experiments with TBADT@silica. These data have been corrected by subtracting any acetophenone detected before irradiation. The green data correspond to yields obtained with silica that had been pre-treated with APTES. The inset shows the effect of APTES on the rate constant for the decay of ^3^DT* phosphorescence in acetonitrile following 355 nm excitation and monitored at 1270 nm; the slope corresponds to the bimolecular rate constant for ^3^DT* quenching by APTES (see Figure S3).

The change in yields over time fits reasonably well with a linear equation. For instance, the same data for TBADT@silica, as shown in Figure 4, is plotted against time in Figure 5. However, in this case, we have subtracted any acetophenone detected at time zero to make both samples more comparable. Both sets have been fitted together with a correlation factor > 0.999. Additionally, the same graph includes data for a single set in which TBADT had been deposited on commercial silica pre-treated with APTES. The resulting slope is approximately four times smaller, indicating that APTES reduces the photochemical reactivity of DT on silica.

We attribute the adverse effect of APTES to its ability to quench the NIR phosphorescence from the triplet state of DT [6,18]. To test this hypothesis, we conducted time-resolved studies on the quenching of the NIR phosphorescence of ^3^DT* by TBADT, as depicted in the inset of Figure 5. These experiments resulted in a quenching rate constant, *k*_Q_, of 2 × 10^9^ M^−1^s^−1^, which is extremely fast. It is not surprising that some quenching occurs when DT and APTES are adjacent on the silica surface. For more details about these experiments, please refer to the Supplementary Information. Figure S3 provides the standard treatment for graphs such as that in the inset in Figure 5.

TiO_2_, a well-known oxidation catalyst, prompted us to investigate whether the simultaneous excitation of electrons to the TiO_2_ conduction band could aid the process. However, our findings indicated that this was not the case, suggesting that the TiO_2_ sun protector properties dominate its role in this system [20].

Several control experiments were conducted, including some using pristine supports, which are detailed in the Supplementary Information. Generally, the yields of acetophenone were low, typically below 10% after a 24 h of UVA irradiation. We also note that the generation of free radicals, including photoreduction of acetophenone [17], can lead to the formation of peroxyl radicals, which can also promote the oxidation of 1-phenylethanol.

#### 2.2.1. Cyclohexanol Sample UVA Irradiation Using TBADT on Different Supports

**Figure 6:**
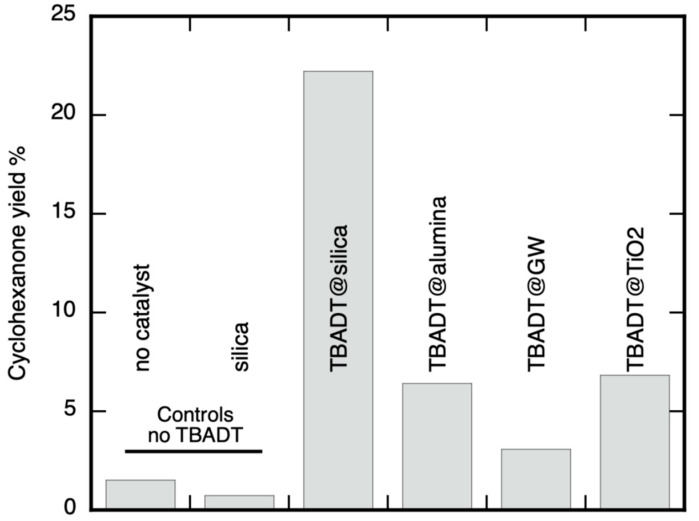
UVA irradiation of cyclohexanol (0.1 M) in chloroform for 24 h under oxygen using various materials. The two columns on the left are control experiments with no catalyst or only silica.

The oxidation of cyclohexanol was investigated to assess the performance of our novel photocatalysts beyond the aromatic alcohols discussed earlier. These studies are similar to those depicted in Figure 4 and resulted in the reaffirmation of TBADT@silica as the preferred catalyst, as illustrated in Figure 6.

Clearly cyclohexanol is less reactive than 1-phenylethanol. This is likely due to a weaker association with the catalytic site, as π-systems usually facilitate this process. In addition, the C-H bond dissociation energies for 1-ohenylethanol and cyclohexanol are reported as 85.1 and 92.8 kcal/mol [21], respectively, thus favoring the former. Higher yields can be obtained by prolonged irradiation (see Supporting Information). Comparative rates for 1-phenylethanol and cyclohexanol show initial rates of 1.1% per hour for cycloxexanone formation and 2.8% per hour for acetophenone.

#### 2.2.2. Cyclohexanol Irradiations at 280 nm

Several experiments were conducted using a 280 nm LED strip as a light source, illuminating quartz vials. The spectrum of this LED illuminator is provided in the Supplementary Information. The highest yield observed was approximately 20% for TBADT@silica. However, in all cases, including the direct photolysis without a catalyst, numerous unidentified products were detected, with up to 20 in this control experiment. Consequently, further studies in this spectral region were not pursued.

#### 2.2.3. Exploratory Studies Using NaDT

As we commenced this work, we hypothesized that depositing NaDT on supports using acetonitrile as the primary solvent, with a small amount of water, while employing organic solvents for the actual experiments, would be advantageous. This approach was based on the assumption that the utilization of “orthogonal solubilities” would minimize any leaching concerns. However, the best yield obtained for 1-phenylethanol using NaDT@silica was only 5.7%, which is almost an order of magnitude lower compared to TBADT@silica (as depicted in Figure 4). Moreover, the leaching of TBADT was not a significant issue, and catalysts based on TBADT could be readily recovered and reused. Consequently, the NaDT studies are regarded as exploratory in nature. Interestingly, APTES did not significantly reduce the yields in the case of NaDT@silica.

While our ICP studies faced reproducibility problems, likely due to acid digestion problems, all indications are that with the protocol used, TBADT incorporation led to tungsten loading roughly three times higher than in the case of NaDT.

#### 2.2.4. Exploratory Studies Using Black-TiO_2_ as Support

Several experiments were conducted using black TiO_2_ as a support for TBADT. The results for UVA and visible light are summarized in the last four entries of Table S1. Conversions after 24 h were consistently within the 5 to 8% range, regardless of the type of light used or whether TBADT was included. This suggests that TBADT is not an improvement over other materials, although it indicates that some oxidations may be possible under visible light conditions. It is likely that the strong absorption of black TiO_2_ itself prevents TBADT from competing as a light absorber.

## 3. Materials and Methods

### 3.1. Sources of Materials

#### 3.1.1. Commercial Materials

All chemicals were purchased from Sigma Aldrich (St. Louis, MO, USA) and used as received unless otherwise stated. They were of analytical pure grade (99%) and used without further purification. This was confirmed as per the manufacturer’s specifications. 3-aminopropyl-functionalized silica gel (40–63 µm and has 1 mmol/g NH_2_ loading) and (3-aminopropyl)triethoxysilane (APTES) were purchased from Sigma-Aldrich (St Louis, MO, USA); this material contains 98% (3-aminopropyl)triethoxysilane.

TiO_2_ P25, a technical-grade powder, was purchased from Univar Canada (Richmond, BC, Canada); this material contains mostly the anatase form of TiO_2_, with some rutile present. Activated alumina oxide (acidic, Brockmann, 58 Å pore size, pH 4.5 ± 0.5 in H_2_O), silicon dioxide nanopowder (1–20 nm particle size, 99.5% trace metals basis), and non-treated glass wool were all obtained from Sigma-Aldrich and used as received.

#### 3.1.2. Preparation of NaDT and TBADT

While both materials are commercially available, their prices per mol are exorbitant, and their synthesis is relatively simple. We chose two materials with different counterions to achieve contrasting solubilities, ensuring their compatibility for incorporation on supports using aqueous or organic solvents.

TBADT was synthesized using tetrabutylammonium bromide and sodium tungstate dihydrate, following the method published earlier [6]. First, 2.4 g (50 mM) of tetrabutylammonium bromide and 5.0 g (101 mM) of sodium tungstate dihydrate were each dissolved in 150 mL of deionized water and heated to 90 °C with continuous stirring. The pH of each solution was adjusted to 2 by adding 11.6 M concentrated hydrochloric acid dropwise. Subsequently, the tetrabutylammonium bromide solution was added to the sodium tungstate solution while stirring. Upon addition, a white suspension formed immediately. The mixture was stirred at 90 °C for 30 min. After this period, the solution was cooled to ambient temperature in a fume hood, and then the TBADT was separated by vacuum filtration. The resulting solid was placed in an oven at 120 °C for 2 h. The powder obtained was transferred to a 100 mL Erlenmeyer flask containing DCM (20 mL of solvent per gram of solid) and stirred vigorously for 1 h. Afterward, the mixture was filtered and the solid was washed with DCM. Finally, the powder was dried in an oven at 120 °C overnight.

To synthesize sodium decatungstate [22], NaDT, two separate 1 L Erlenmeyer flasks were prepared, each with a stir bar. One flask contained 44 g (133 mmol) of sodium tungstate dihydrate dissolved in 250 mL of deionized water. The other flask held 250 mL of 0.1 M hydrochloric acid. Both flasks were heated at 95 °C and stirred until this temperature was reached. Once both solutions were at the desired temperature, the hot HCl solution was slowly added to the sodium tungstate solution. The combined mixture was stirred at 95 °C for 60 s. The resulting pale green solution was quickly transferred into a 2 L Erlenmeyer flask that had been chilled in an ice bath. Stirring continued as the solution cooled. When the temperature dropped to 10 °C, 180 g (6.2 M) of sodium chloride was added. The mixture was then placed in an ice bath and stirred at 0 °C for one hour, forming a milky suspension. This suspension was filtered under vacuum, and the solid was washed sequentially with cold deionized water, ethanol, and diethyl ether (30 mL each). The solid was then dried in an oven at 120 °C for 30 min and subsequently transferred to a 100 mL Erlenmeyer flask containing a stir bar. The flask was placed in an acetonitrile bath at 80 °C for one hour. The solid was filtered and washed with ethanol, then placed in an oven at 120 °C.

### 3.2. Modification of Supports

#### 3.2.1. APTES@Glass Wool

Non-silanized glass wool (~1.3 g) was immersed in 6 M HCl and stirred for 72 h. Subsequently, it was washed with deionized water until its pH reached 6. The acid-treated glass wool was then functionalized with 3-aminopropyltriethoxysilane (APTES) by dispersing it in 110 mL of HPLC-grade toluene containing 1.1 mL of APTES in a round-bottom flask (1% concentration of APTES in toluene). The mixture was refluxed at 130 °C in an oil bath under continuous stirring overnight. The following morning, the mixture was removed from the oil bath and cooled to room temperature. The suspension was stirred for an additional 6 h. The treated glass wool was washed three times each with HPLC-grade toluene and acetone and then placed in a 100 °C oven for 24 h prior to decoration with DT.

#### 3.2.2. Synthesis of Black TiO_2_

Black TiO_2_ was synthesized through a thermal reduction process using ethanol as a reducing agent under vacuum [23]. A 2.4 g measure of P25 TiO_2_ was placed inside a quartz tube and inserted into a tubular furnace. Separately, 10–15 mL of 98% ethanol was transferred to a beaker and immersed in a liquid nitrogen bath until it froze completely (approximately 15 min). The ethanol freezing setup involved placing the beaker in a thermally insulated container filled with liquid nitrogen, which was then transported in a sealed thermal vessel within a Styrofoam box containing ice packs. Once the ethanol was frozen, the beaker was rapidly transferred into the quartz tube containing the TiO_2_. The tube was immediately sealed, and a vacuum was applied using a rotary pump until a pressure of 10^−4^ Torr was reached. After achieving the desired vacuum, the valve was closed, and the pump was turned off.

The furnace was heated to 400 °C at a rate of 5 °C per minute and maintained at this temperature for 3 h. After the heat treatment, the system was cooled to 50 °C at the same rate and then allowed to cool naturally to room temperature under vacuum. Once the ambient temperature was reached, the system was vented to atmospheric pressure by opening the air outlet.

The resulting black TiO_2_ catalyst was washed in situ by flowing deionized water through the quartz tube using a peristaltic pump (Gilson, Inc., Middleton, WI, USA). After washing, the catalyst was dried by connecting the tube to a compressed air outlet until fully dry.

### 3.3. Preparation of DT@support Catalysts

Heterogeneous catalysts were prepared by immobilizing decatungstate on various support materials, such as silica (SiO_2_), alumina (Al_2_O_3_), titanium dioxide (TiO_2_), and glass wool. Decatungstate salt (1.0 g), either sodium decatungstate (NaDT) or tetrabutylammonium decatungstate (TBADT), was first dissolved in a solvent mixture of acetonitrile (60 mL) and deionized water (15 mL) in a 250 mL round-bottom flask equipped with a rotary evaporator. The selected support material (5.0 g) was then added to the solution while the flask was stirred. The resulting suspension was rotated for 3 h to ensure uniform dispersion of the catalyst precursor. Afterward, the solvent was removed by rotary evaporation over 2 h at 30 °C. The resulting solid was collected and dried overnight in an oven at 120 °C to yield the final DT@support catalysts.

### 3.4. Irradiation of the Samples

Each sample was irradiated in a fused quartz crimp vial from Technical Glass Products. The 10 mL vial contained 0.14 g of supported DT, 61 µL of 1-phenylethanol (0.1 M), 39 µL of internal tert-butylbenzene standard (0.05 M), and 5 mL of chloroform. When the loading of TBADT on the support is considered, this corresponds to approximately a 1.4 mol percent catalyst load. The vial was flushed with oxygen for 8 min before irradiation and again for 5 min after 3 h of irradiation. The samples were slowly rotated in a hot dog cooker and irradiated with two UVA lamps (8 W power each) placed on top of them at room temperature; see Figure 3. The samples were analyzed by GC-MS or NMR at various times, always including 0 and 24 h samples. We note that chloroform proved a convenient solvent for GC and NMR analysis; its use is not essential for synthetic applications and can be readily replaced by other solvents, for example, acetonitrile.

### 3.5. Leaching Tests for TBADT@silica

We conducted a leaching test using a TBADT@silica catalyst. We added 0.14 g of the catalyst to 5 mL of chloroform, left it undisturbed in the dark for 24 h, and then transferred the supernatant to a new vial without the catalyst. We added 1-phenylethanol and irradiated it with UVA light. The minimal leaching was confirmed by the small percentage yield of acetophenone.

We repeated the experiment with a different reaction mixture: 0.14 g of TBADT@silica, 5 mL of chloroform containing 0.1 M 1-phenylethanol, and 0.05 M tert-butylbenzene. We irradiated the mixture with UVA light for three hours and then used GC–MS analysis to determine the products. We found 2.2% acetophenone formation. We transferred the supernatant to a fresh quartz vial and irradiated it for another 21 h, resulting in minimal leaching with an increased but small percentage yield of acetophenone (8.5%) but decreased selectivity and the formation of multiple side products.

### 3.6. Tools and Instruments Used for Analysis and Characterization

Diffuse reflectance measurements were performed using an Agilent Cary 7000 UV-Vis-NIR Universal Measurement Spectrophotometer (Agilent, Santa Clara, CA, USA) coupled with an Agilent Praying Mantis accessory. This instrument records F(R) spectra according to Kebelka–Munk analysis [19,24]. Scanning electron microscopy (SEM) images were collected on a JSM-7500F field emission scanning electron microscope from JEOL Ltd. (Tokyo, Japan). All laser flash photolysis (LFP) experiments were performed using a customized LFP-111 laser flash photolysis system (Luzchem Inc., Ottawa, ON, Canada) coupled with a Surelite Nd-YAG laser with the third harmonic wavelength of 355 nm (<20 mJ pulse^−1^). Samples had an absorbance of ∼0.3 at the laser wavelength, and a quartz cuvette with a 1 cm pathway was used for LFP experiments. Mass Spectra (GC-MS) or Nuclear Magnetic Resonance (NMR) were used to analyze the products. The instrument used for NMR spectroscopy was a Bruker Avance 300 MHz NMR (Madison, WI, USA). GC-MS was performed using mass spectrometry on an Agilent 6890-N gas chromatograph with an Agilent 5973 mass selective detector. Inductively coupled plasma optical emission spectroscopy (ICP-OES) measurements were carried out using an Agilent 5110 ICP-OES spectrometer (Agilent Technologies, Santa Clara, CA, USA). This instrument provides simultaneous multi-element analysis with axial and radial plasma viewing for high sensitivity and a wide dynamic range. X-ray diffraction (XRD) experiments were performed using a powder X-ray diffractometer, Bruker D8 Endeavor (Bruker, Billerica, MA, USA), with a diffracted beam monochromator Cu Kα source (40 kV-40 mA). All patterns were recorded from 2Θ = 5° to 90°, in steps of 0.02°. The Brunauer–Emmett–Teller (BET) measurements were performed at Carleton University using a Nova 800 physisorption analyzer (Anton Paar, Graz, Austria), equipped with two stations (four ports) available for simultaneous operation in both degas and analysis chambers. For sample preparation, the instrument employs vacuum degassing, where the sample is heated to 90 °C at a ramp rate of 5 °C/min and held for 600 min to remove adsorbed impurities. During analysis, the instrument performs a full nitrogen isotherm adsorption–desorption profile at 77.35 K, with p/p_0_ ranging from 0 to 1. Measurements are carried out using a 9 mm cell with a filler rod, under helium void volume calibration. The thermal delay is set to 180 s. This setup enables precise surface area determination via multipoint BET, as well as pore size and volume characterization using the NLDFT method on nitrogen at 77 K. From these analyses, the instrument provides BET surface area values, total pore volume, specific surface area from DFT methods, and pore size distribution derived from the desorption branch of the isotherm.

## 4. Conclusions

Decatungstate, an exceptional photo-oxidation catalyst, is renowned for its capacity to catalyze Type I oxidations mediated by radicals and radical ions, but not singlet oxygen. This unique characteristic distinguishes it from other excited triplet states, as it exhibits resilience to oxygen quenching. Typically, catalyst loads, such as 2 mol percent, may not fully reveal the substantial mass load of compounds with molecular weights exceeding 3000, underscoring the critical importance of recovery and reuse. Consequently, the design of heterogeneous catalysts becomes paramount. Our comparative study of various forms of supported decatungstates significantly advances our comprehension of achieving photocatalysis with reusable materials. Furthermore, we believe that our materials possess the potential to facilitate continuous flow photocatalysis in the foreseeable future. Our studies indicate that TBADT@silica is the catalyst of choice. This excellent catalyst is easy to prepare from commercially available precursors.

## Data Availability

The original contributions presented in this study are included in the article/Supplementary Materials. Further inquiries can be directed to the corresponding author.

## Supplementary Materials

The following supporting information can be downloaded at https://www.mdpi.com/article/10.3390/molecules30173597/s1. Spectra of light sources; Laser flash photolysis NIR data; diffuse reflectance of catalysts used; tables of experimental results, GC-MS data, and SEM data; Python code to correct spectra obtained with Cary spectrometers.
