# Supplementary material for: Comparison of Composite Materials Designed to Optimize Heterogeneous Decatungstate Oxidative Photocatalysis
Source: Molecules. 2025 Sep 3;30(17):3597. doi: 10.3390/molecules30173597 (PMC12430545; doi:10.3390/molecules30173597)
Supplement: Supplementary file 1 [file molecules-30-03597-s001.zip › molecules-3824748-supplementary.pdf]

Supplementary Information

Comparison of composite materials designed to optimize heterogeneous decatungstate oxidative photocatalysis

Julia Ong, Benjamin Cajka and Juan C. Scaiano

Department of Chemistry and Biomolecular Sciences, University of Ottawa, Ottawa, Ontario, K1N 6N5, Canada

Table for Contents:

EMISSION SPECTRA OF LIGHT SOURCES 3

LUZCHEM UVA LAMPS 3

VIOLUMAS 280 NM LED STRIP 4

LASER FLASH PHOTOLYSIS DATA OF DT* QUENCHED BY APTES 5

QUENCHING PLOT 5

PHOSPHORESCENCE DECAY TRACES 6

REPRESENTATIVE SEM IMAGES 14

CORRECTION OF DIFFUSE REFLECTANCE SPECTRA VIA WEIGHTED AVERAGING 16

DATA CORRECTING ALGORITHM 16

PYTHON CODE FOR CORRECTING LIGHT CHANGE AT 350 NM AND 420 NM 17

EXAMPLE OF DIFFUSE REFLECTANCE SPECTRA BEFORE AND AFTER CORRECTION 21

DIFFUSE REFLECTANCE SPECTRA OF CATALYSTS 22

ICP RESULTS OF CATALYST FOLLOWING ACID DIGESTION PREPARATION 32

XRD RESULTS OF CATALYSTS 33

TABLE S1: CRYSTALLITE SIZE CALCULATIONS FOR TBADT, SITBADT AND RECYCLED SITBADT, ALL USING THE SCHERRER EQUATION 37

TABLES SHOWING THE DATA ON WHICH VARIOUS GRAPHS ARE BASED 38

TABLE S2: YIELDS (%) OBTAINED UNDER UVA IRRADIATION FOR 1-PHENYLETHANOL SAMPLES 38

TABLE S3: YIELDS (%) OBTAINED UNDER UVA AND 280 NM IRRADIATION FOR CYCLOHEXANOL SAMPLES 40

BET RESULTS FOR TBADT ON SELECTED SUPPORTS 41

PRODUCT STUDIES 42

REPRESENTATIVE GC-MS CHROMATOGRAM 42

Emission Spectra of Light sources

Luzchem UVA lamps

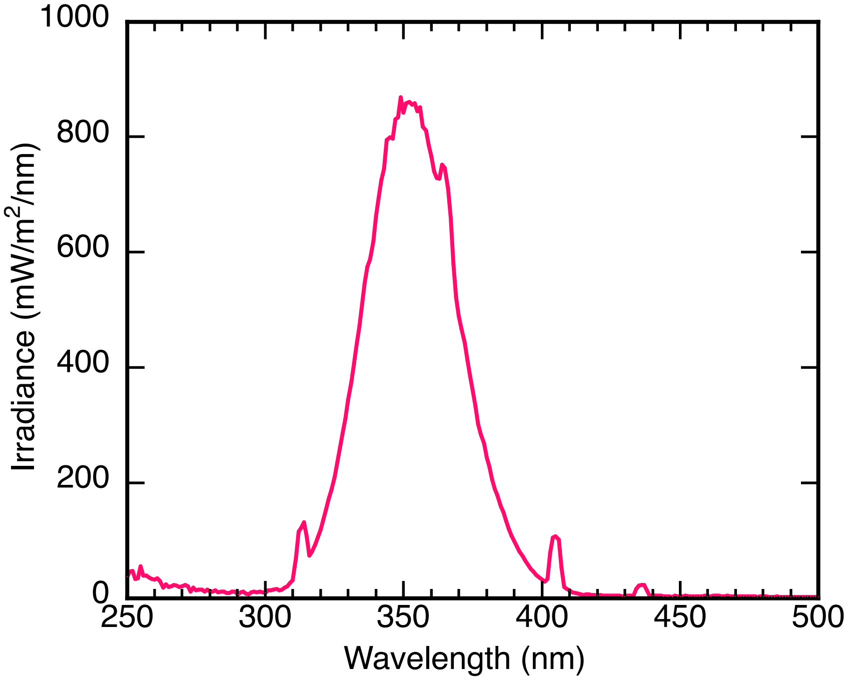

Figure S1: Emission spectra of two UVA lamps (8 W) in a Luzchem Expo panel. Irradiance intensity measurements were taken directly below the light source, with a recorded average intensity of 37.9 W/m², integrated between 300 and 450 nm.

Violumas 280 nm LED strip

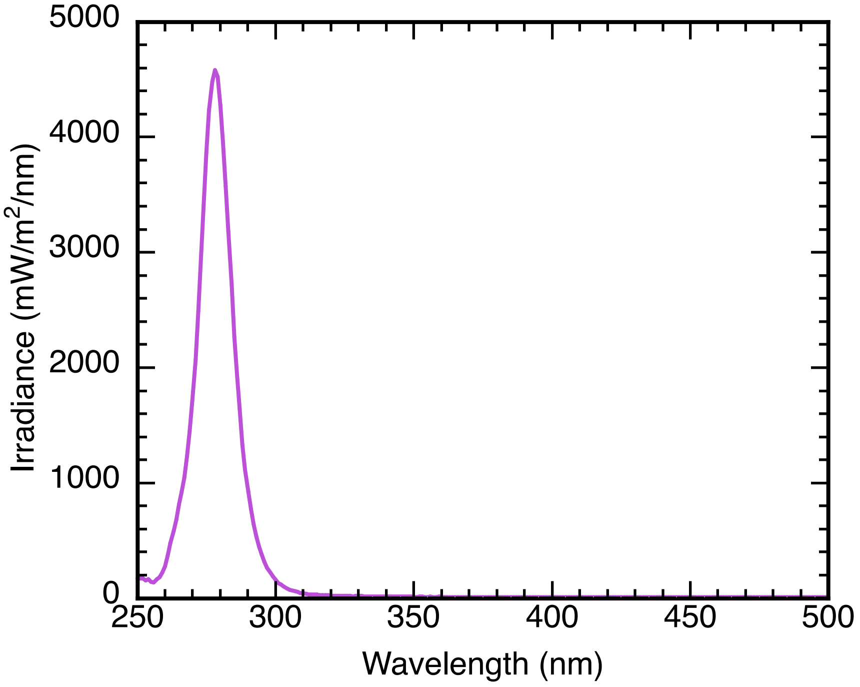

Figure S2: Emission spectra of 280 nm light from a Violumas 12 LED strip. Irradiance intensity measurements were taken directly below the light source, with recorded average intensity of 89.9 W/m², integrated between 250 and 330 nm

Laser flash photolysis data of DT* Quenched by APTES

Quenching plot

The plot below is the same one as in the inset in Figure 5, but with a few more data points. Each point is based on one of the NIR decay graphs in the following section.

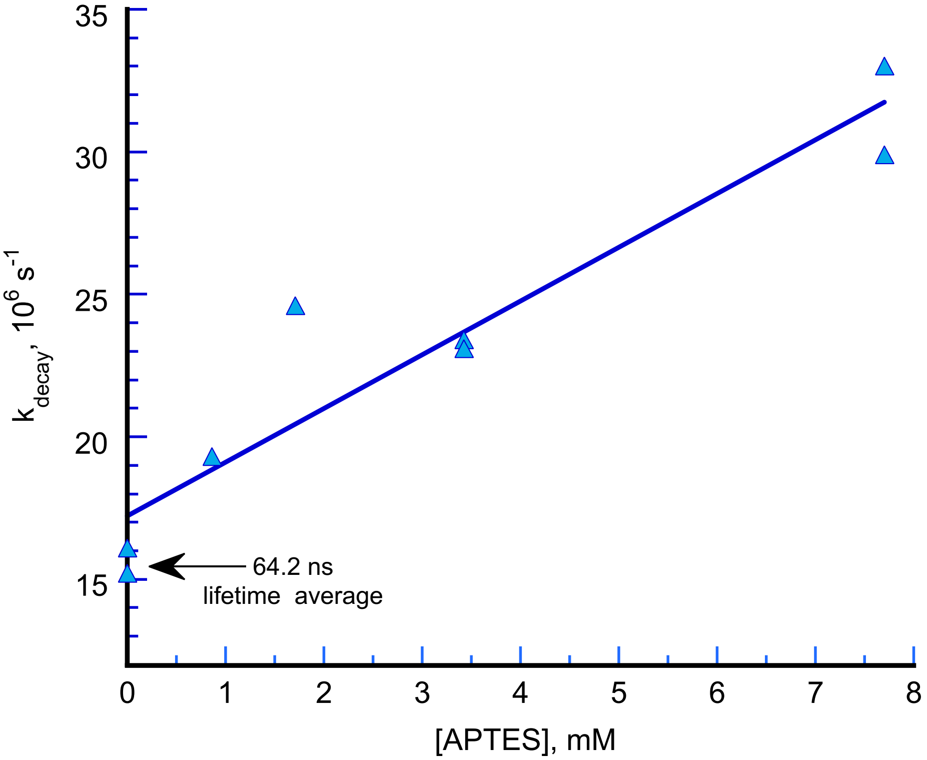

Figure S3: Effect of APTES on the rate constant for the decay of ^{3}DT* phosphorescence in acetonitrile following 355 nm excitation and monitored at 1270 nm

Phosphorescence decay traces

Each decay trace below was monitored at 1270 nm following laser excitation at 355 nm and after various APTES additions.

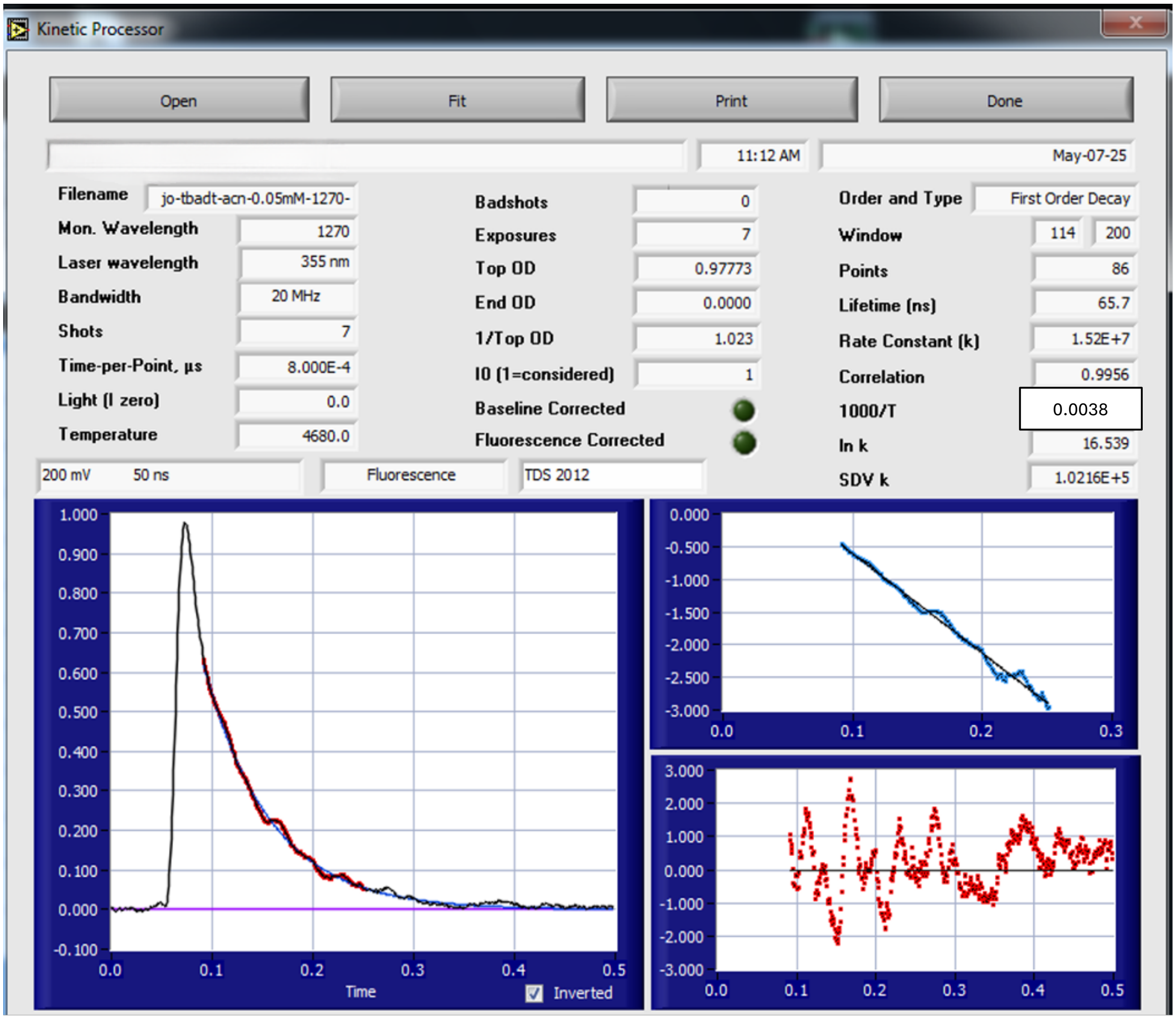

Figure S4: Phosphorescence decay of decatungstate from TBADT in acetonitrile monitored at 1270 nm for 0.1 mM TBADT in chloroform. Instrument bandwidth was bandwidth of 20 MHz, and the excitation laser was at 355 nm.

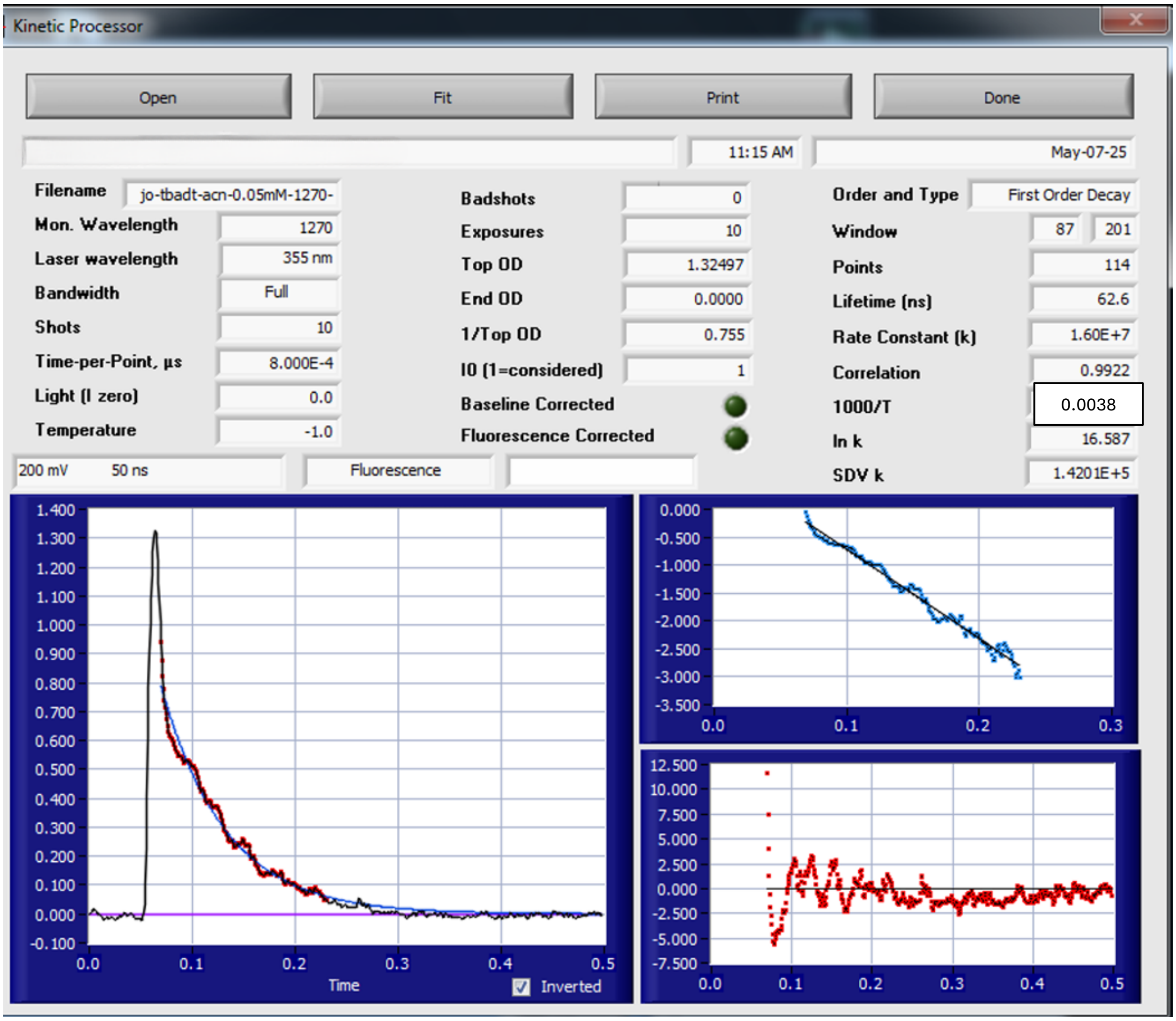

Figure S5: Phosphorescence decay of decatungstate from TBADT in acetonitrile monitored at 1270 nm for 0.1 mM TBADT in chloroform. Instrument bandwidth was full bandwidth, and the excitation laser was at 355 nm.

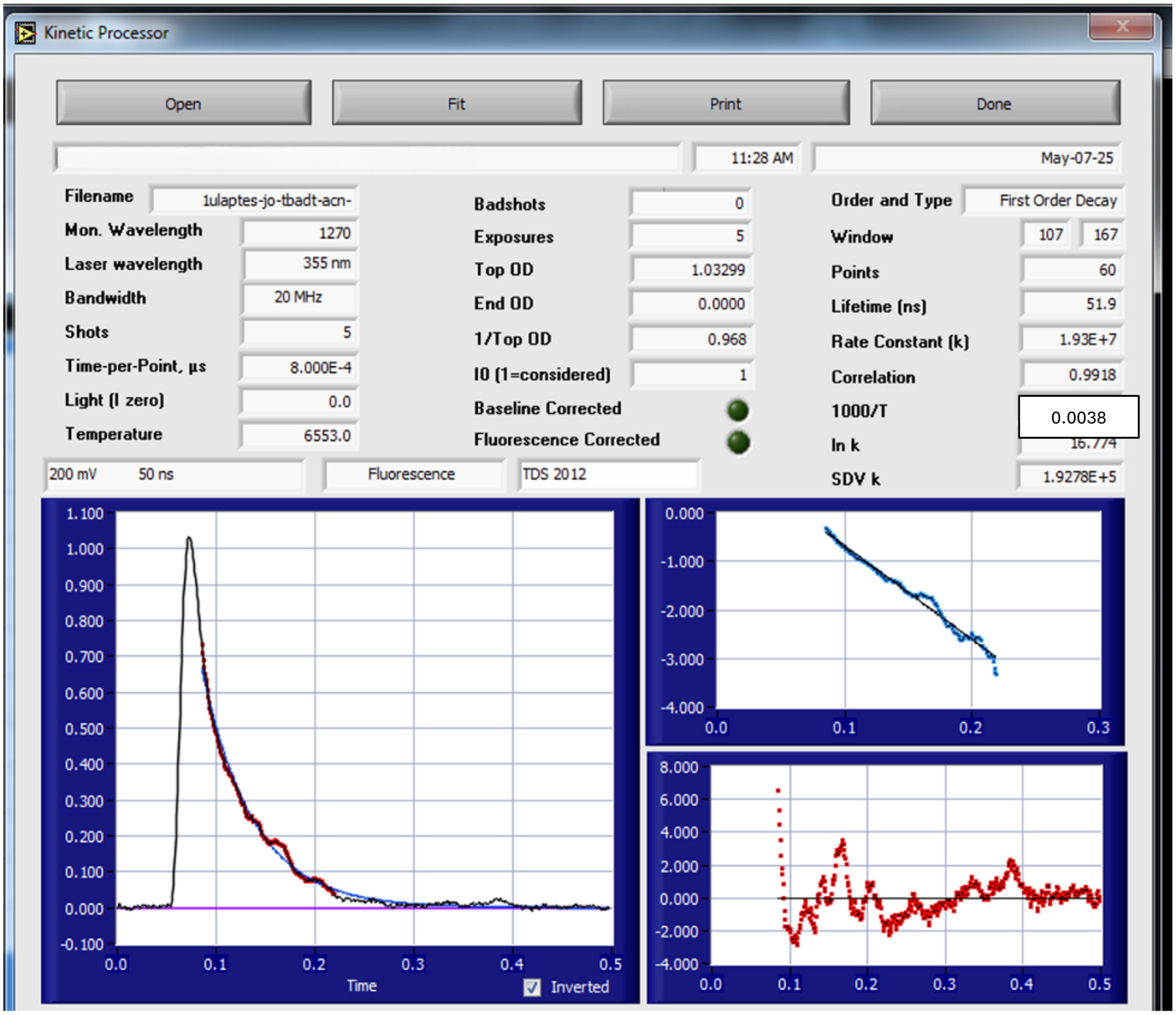

Figure S6: Phosphorescence decay of decatungstate from TBADT in acetonitrile monitored at 1270 nm for 0.1 mM TBADT in chloroform after the addition of 0.86 mM APTES. Instrument bandwidth was bandwidth of 20 MHz, and the excitation laser was at 355 nm.

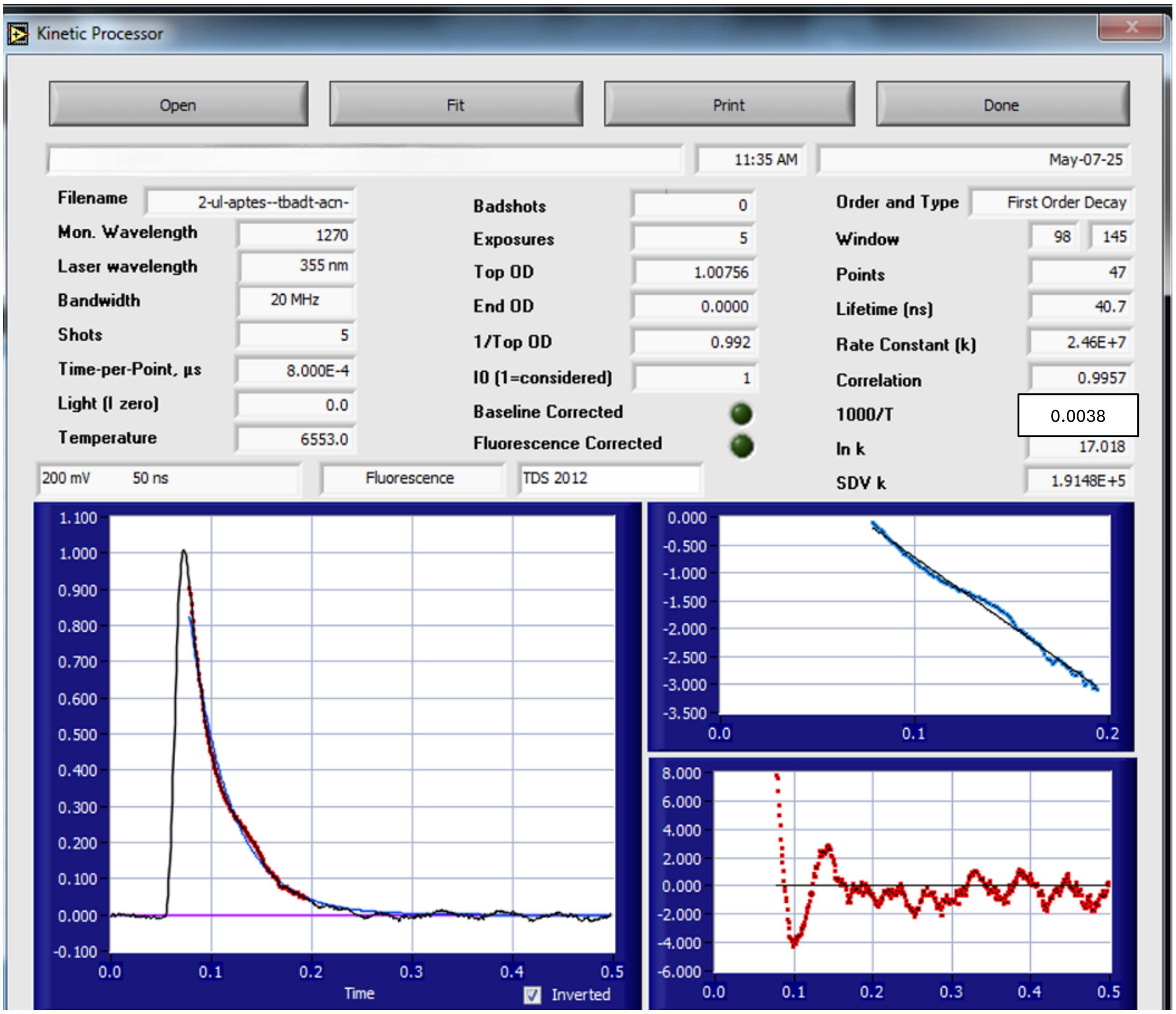

Figure S7: Phosphorescence decay of decatungstate from TBADT in acetonitrile monitored at 1270 nm for 0.1 mM TBADT in chloroform after the addition of 1.71 mM APTES. Instrument bandwidth was bandwidth of 20 MHz, and the excitation laser was at 355 nm.

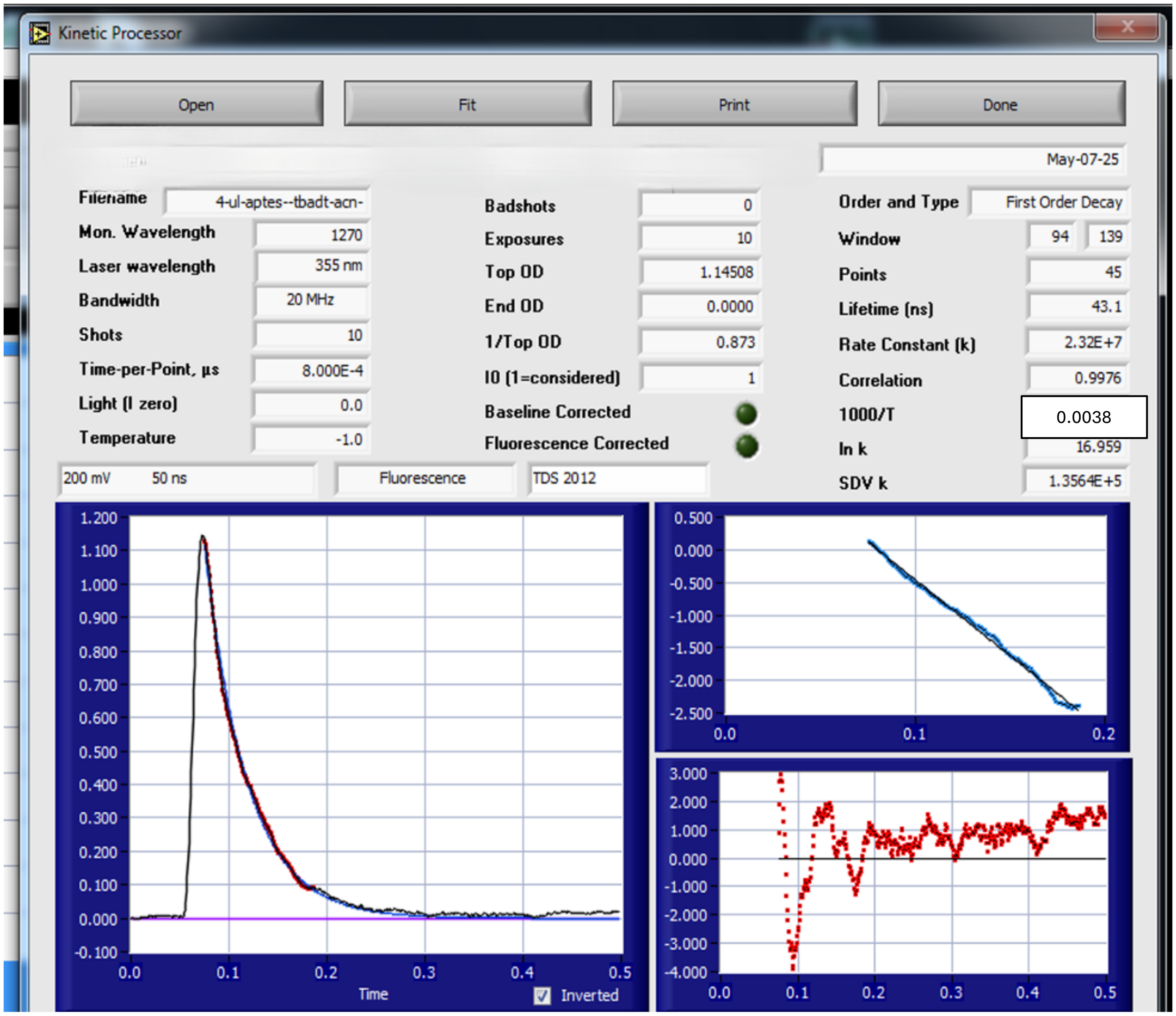

Figure S8: Phosphorescence decay of decatungstate from TBADT in acetonitrile monitored at 1270 nm for 0.1 mM TBADT in chloroform after the addition of 3.42 mM APTES. Instrument bandwidth was bandwidth of 20 MHz, and the excitation laser was at 355 nm.

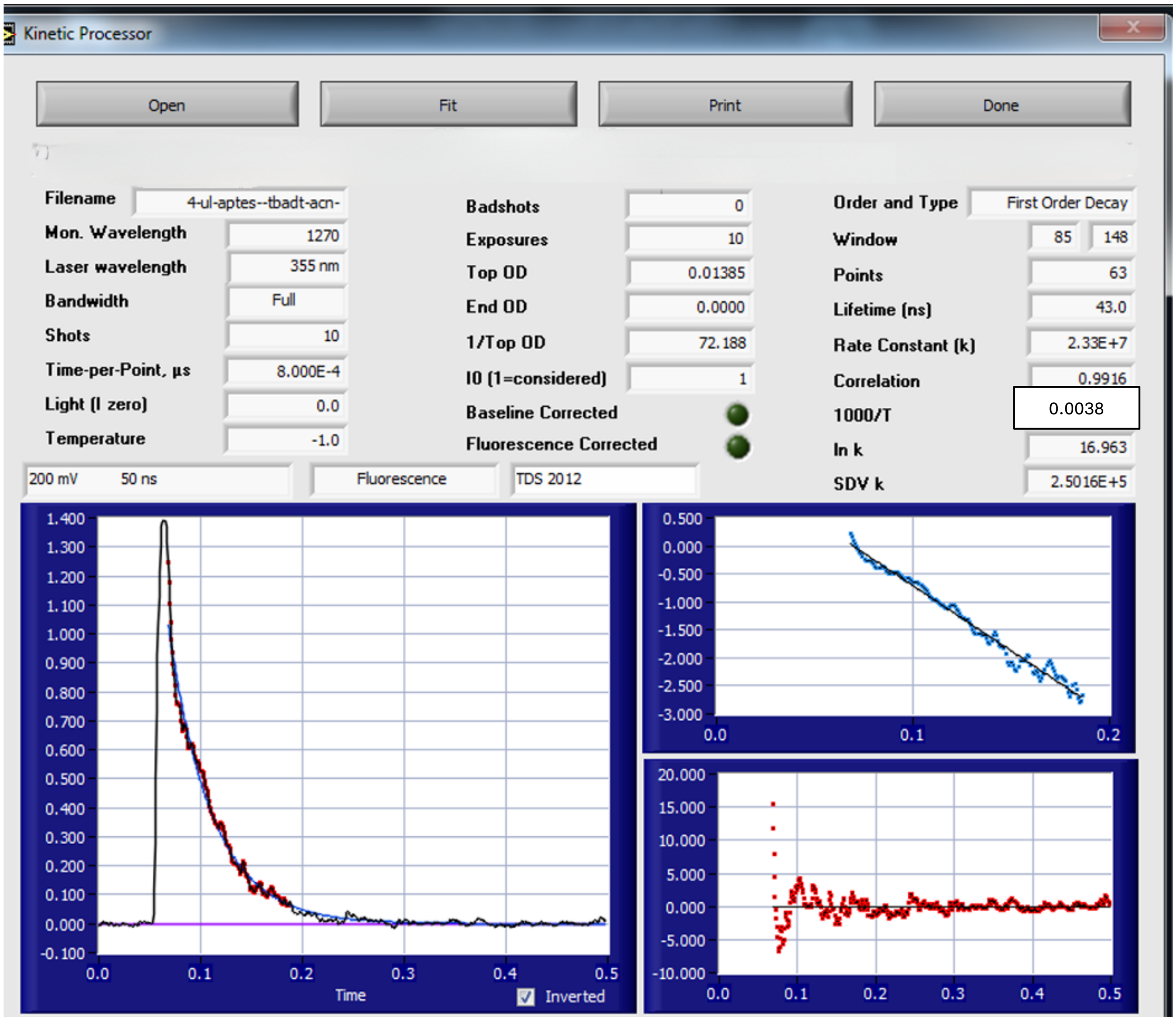

Figure S9: Phosphorescence decay of decatungstate from TBADT in acetonitrile monitored at 1270 nm for 0.1 mM TBADT in chloroform after the addition of 3.42 mM APTES. Instrument bandwidth was full bandwidth, and the excitation laser was at 355 nm.

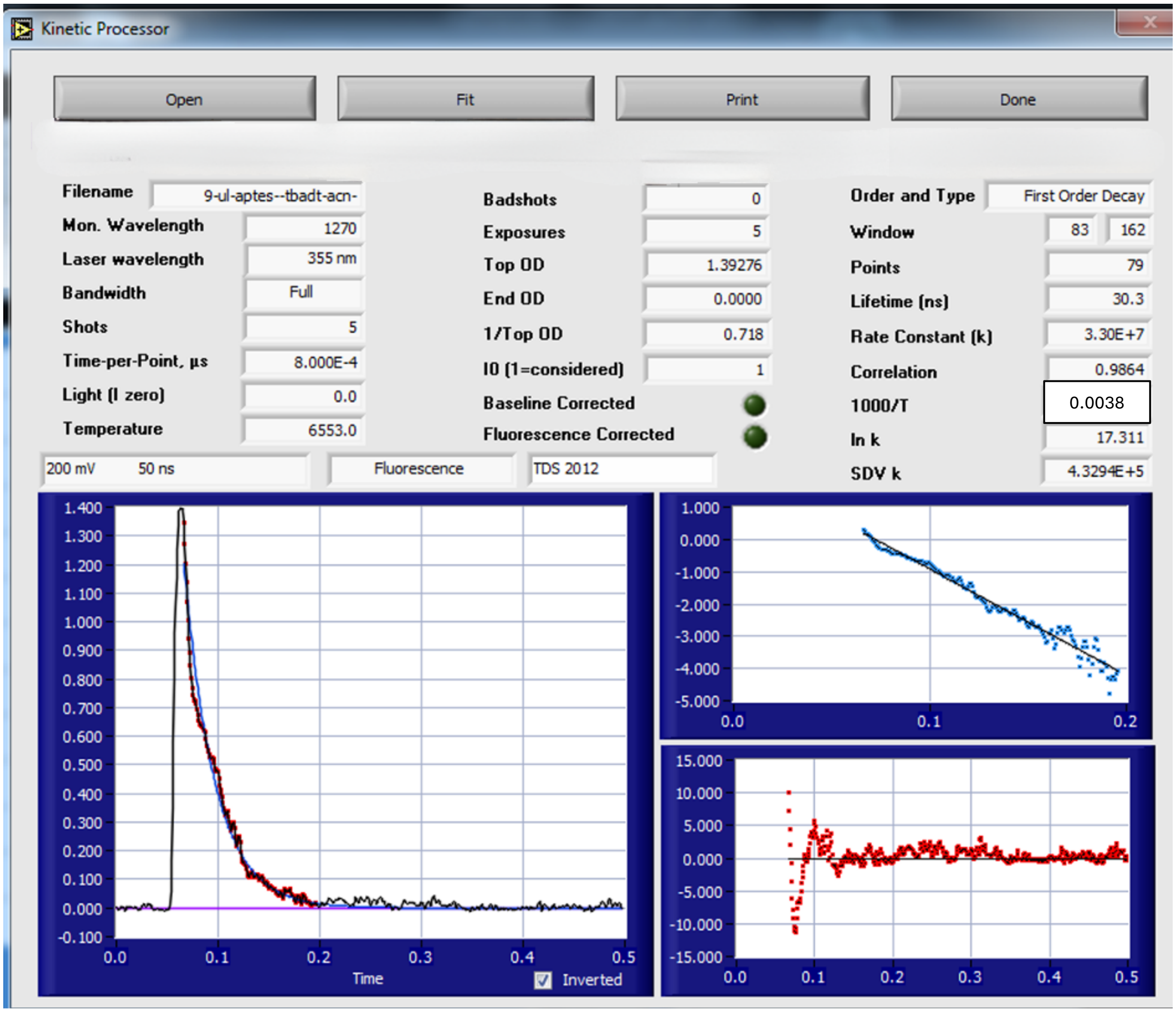

Figure S10: Phosphorescence decay of decatungstate from TBADT in acetonitrile monitored at 1270 nm for 0.1 mM TBADT in chloroform after the addition of 7.7 mM APTES. Instrument bandwidth was full bandwidth, and the excitation laser was at 355 nm.

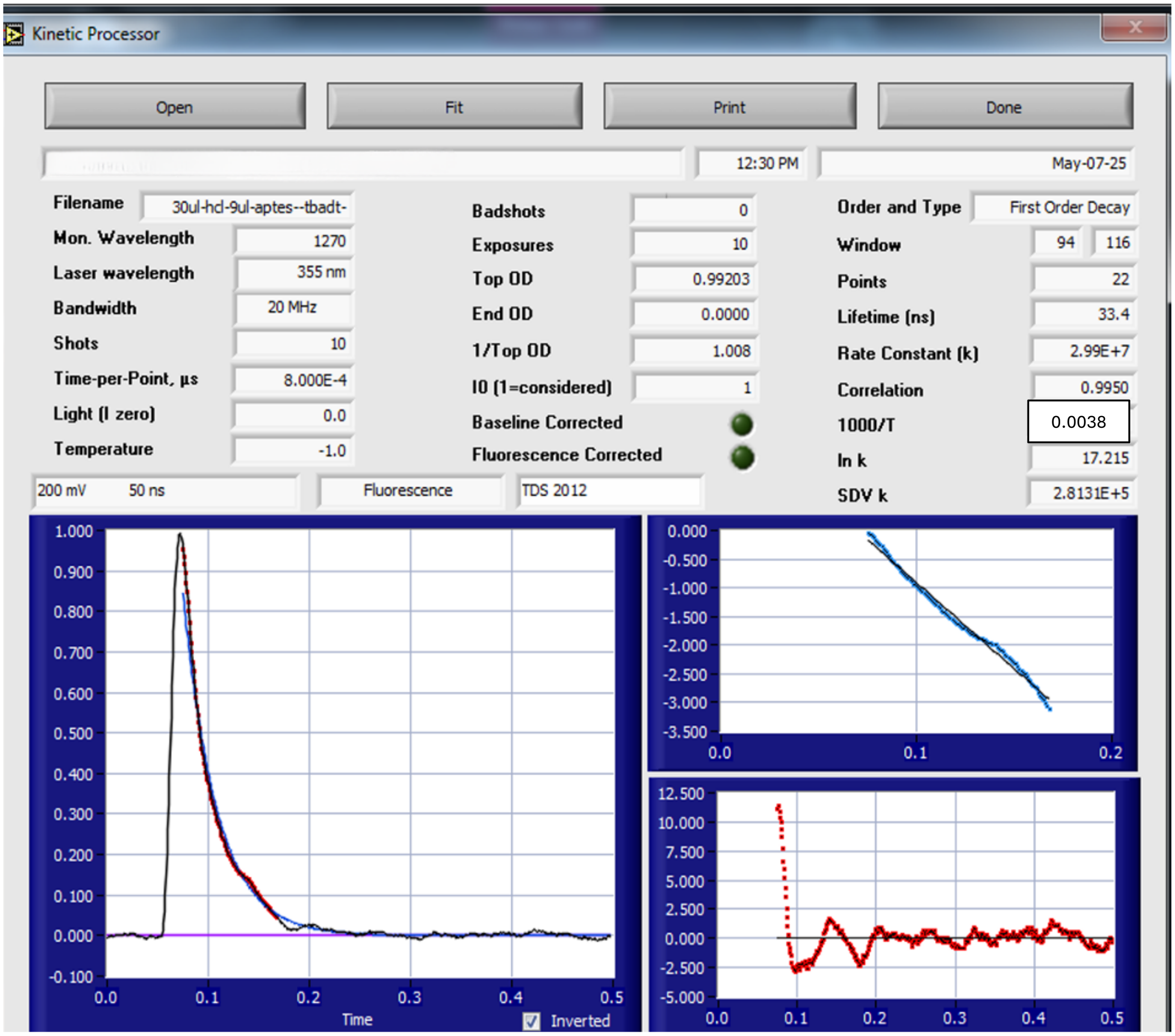

Figure S11: Phosphorescence decay of decatungstate from a 0.1 mM TBADT solution in acetonitrile was monitored at 1270 nm after the sequential addition of 7.7 mM APTES and 30 µL of HCl (0.348 mmol), to assess whether the quenching effect could be reversed. The instrument had a bandwidth of 20 MHz, and the excitation laser wavelength was 355 nm.

Representative SEM Images

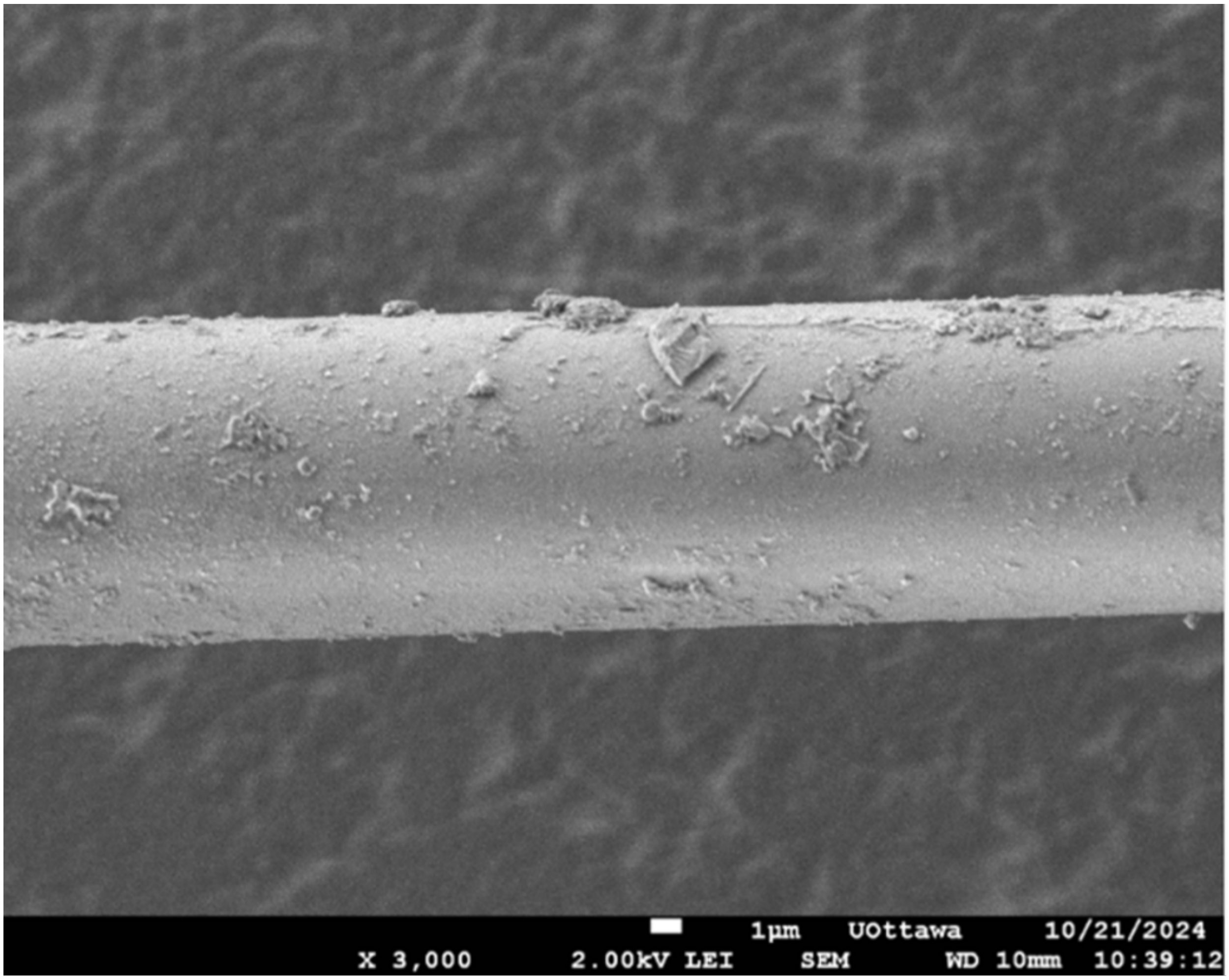

Figure S12: SEM image of DTGW made with HCl treatment, showing the DT dispersion along the GW surface, and monolayer coverage of APTES.

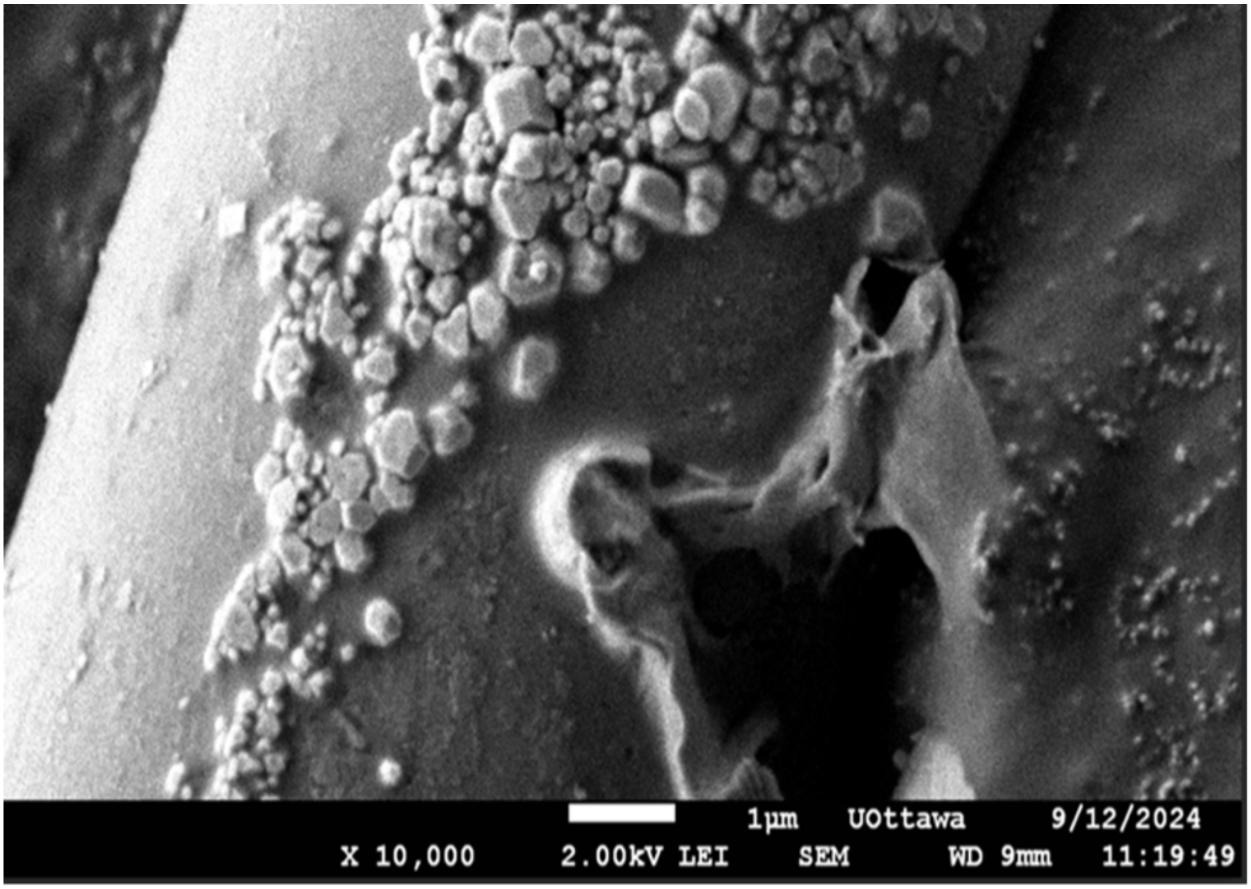

Figure S13: SEM image of DT@GW, showing the DT cluster size and crystal shape.

Correction of Diffuse Reflectance Spectra via Weighted Averaging

Cary spectrometers frequently show a “jump” in the data in the region where the lamp changes, typically between UV and visible regions. In our laboratory this lamp change is usually performed at 350 nm or 420 nm. We have developed software that will produce a reliable smooth spectrum by giving opposite priority to spectra run twice with different lamp change wavelength. For example, when 350 nm or 420 nm are the chosen wavelengths, data in the vicinity of 350 nm prioritizes the spectrum where the lamp change is at 420 nm and vice versa. A smooth weighted function is used between 351 and 419 nm.

Data correcting algorithm

The following is the algorithm used by the Python code shared below:

- The lamp change wavelengths are λ_1 and λ_2, with $\lambda_1 < \lambda_2$.
- The two data sets acquired are S_1 and S_2, associated with λ_1 and λ_2, and can represent absorbance or reflectance F(R). Importantly, the data sets S_1 and S_2 must have the reference acquired with the same lamp change wavelength as used for data acquisition.
- Weight factors w_1 and w_2 are assigned to the data sets S_1 and S_2, respectively.
  - For $\lambda < \lambda_1$ the values are w_1 = 0 and w_2 = 1
  - For $\lambda > \lambda_2$ the values are w_1 = 1 and w_2 = 0
  - For $\lambda < \lambda_2$ and $\lambda > \lambda_1$; that is the region between lamp changes, then the weight factors are calculated with equations 1 and 2:

$$w_2 = \frac{\lambda - \lambda_1}{\lambda_2 - \lambda_1} \qquad (1)$$

$$w_1 = 1 - w_2 \qquad (2)$$

- Then, the resulting signal, S_λ is given by equation 3:

$$S_\lambda = w_1 \times S_1 + w_2 \times S_2 \qquad (3)$$

The choice of lamp changes is user defined. Note that the reference spectrum should be run at the same wavelength as the sample spectrum. The Python code implementing this algorithm is provided below as Figure S14.

Python Code for correcting light change at 350 nm and 420 nm

(This code can also be used for wavelength changes at other wavelengths)

```
import pandas as pd  # Library for handling tabular data (like Excel)
import numpy as np   # Library for numerical operations
import matplotlib.pyplot as plt  # Library for creating plots

# === USER INPUT ===

# Enter your file names below.
# These should be CSV files with two columns: Wavelength and F(R)
file_high = "Enter file name for higher wavelength light.csv"  # e.g.,
file with reflectance measured at 420 nm
file_low = "Enter file name for lower wavelength light.csv"    # e.g.,
file with reflectance measured at 350 nm

# Define transition range where the light source changes(user input)
lambda_low = 350   # Start of transition (nm)
lambda_high = 420  # End of transition (nm)

# Define custom plot range (user input)
plot_min = 200     # Minimum wavelength for plotting (nm)
plot_max = 500     # Maximum wavelength for plotting (nm)

# === LOAD DATA ===
# Read wavelength and F(R) data from both files.
# We skip the first row (usually header) and rename the columns.
df_high = pd.read_csv(file_high, skiprows=1, usecols=[0, 1],
names=["Wavelength", "FR_High"])
df_low = pd.read_csv(file_low, skiprows=1, usecols=[0, 1],
names=["Wavelength", "FR_Low"])

# === CONVERT TO NUMERIC ===
# Ensure all values are numbers (in case there are missing or invalid
values)
df_high["Wavelength"] = pd.to_numeric(df_high["Wavelength"],
errors='coerce')
df_high["FR_High"] = pd.to_numeric(df_high["FR_High"], errors='coerce')
df_low["Wavelength"] = pd.to_numeric(df_low["Wavelength"],
errors='coerce')
df_low["FR_Low"] = pd.to_numeric(df_low["FR_Low"], errors='coerce')

# === DROP NaNs ===
# Remove rows with missing values
df_high.dropna(inplace=True)
df_low.dropna(inplace=True)
```

```
# === ROUND WAVELENGTHS ===
# Round wavelengths to 2 decimal places to ensure accurate merging
df_high["Wavelength"] = df_high["Wavelength"].round(2)
df_low["Wavelength"] = df_low["Wavelength"].round(2)

# === MERGE ON WAVELENGTH ===
# Combine the two datasets based on matching wavelength values
df = pd.merge(df_high, df_low, on="Wavelength")

# === DEFINE WEIGHTS ===
# This function calculates how much each original spectrum contributes to
the final corrected value
# It uses the custom-defined transition range from lambda_low to
lambda_high
def compute_weights(wavelength):
    weight_low = np.zeros_like(wavelength, dtype=float)

    # Apply a linear transition for wavelengths between lambda_low and
lambda_high
    cond_low = [
        (wavelength <= lambda_low),                          # below
transition → only high spectrum used
        (wavelength > lambda_low) & (wavelength < lambda_high),  # mix of
both
        (wavelength >= lambda_high)                          # above
transition → only low spectrum used
    ]
    values_low = [
        0,                                                   # weight for
low spectrum is 0 below transition
        (wavelength - lambda_low) / (lambda_high - lambda_low),  #
linearly increase from 0 to 1
        1                                                    # weight for
low spectrum is 1 above transition
    ]
    weight_low = np.select(cond_low, values_low)
    weight_high = 1 - weight_low  # complementary weights for high
spectrum

    return weight_low, weight_high

# === APPLY WEIGHTS ===
# Use the computed weights to blend both spectra into a corrected one
df["Weight_Low"], df["Weight_High"] =
compute_weights(df["Wavelength"].values)
```

```
# === CORRECTED SPECTRUM ===
# Create the corrected spectrum by weighted averaging of the two inputs
df["Corrected"] = df["Weight_High"] * df["FR_High"] + df["Weight_Low"] *
df["FR_Low"]

# === FILTER AND SORT FOR 200-800 nm ===
# Only keep data within the visible range and sort by wavelength
df_filtered = df[(df["Wavelength"] >= 200) & (df["Wavelength"] <=
800)].sort_values(by="Wavelength")

# === SAVE FULL CSV ===
# Save all relevant data to a CSV file
df_filtered.to_csv("All-Spectra.csv", index=False)

# === SAVE ONLY WAVELENGTH & CORRECTED ===
# Save just the wavelength and corrected spectrum values to a simpler CSV
df_corrected_only = df_filtered[["Wavelength", "Corrected"]]
df_corrected_only.to_csv("corrected_only.csv", index=False)

# === FILTER FOR PLOTTING (user-defined range) ===
# Only plot the visible region up to the user-specified maximum
df_plot = df_filtered[(df_filtered["Wavelength"] >= plot_min) &
(df_filtered["Wavelength"] <= plot_max)]

# === PLOT ALL SPECTRA ===
plt.figure(figsize=(10, 6))  # Set figure size
plt.plot(df_plot["Wavelength"], df_plot["FR_High"], label=f"{lambda_high}
nm Spectrum", color="red", alpha=0.5)
plt.plot(df_plot["Wavelength"], df_plot["FR_Low"], label=f"{lambda_low} nm
Spectrum", color="green", alpha=0.5)
plt.plot(df_plot["Wavelength"], df_plot["Corrected"], label="Corrected
Spectrum", color="blue", linewidth=2)
plt.xlabel("Wavelength (nm)")
plt.ylabel("F(R)")  # Reflectance
plt.legend()
plt.grid(True)
plt.tight_layout()
plt.savefig(f"All_Spectra_plot_{plot_min}-{plot_max}nm.png")  # Save
figure
plt.show()  # Display the plot

# === PLOT ONLY CORRECTED SPECTRUM ===
plt.figure(figsize=(10, 6))
plt.plot(df_plot["Wavelength"], df_plot["Corrected"], label="Corrected
Spectrum", color="blue", linewidth=2)
plt.xlabel("Wavelength (nm)")
```

```
plt.ylabel("F(R)")
plt.legend()
plt.grid(True)
plt.tight_layout()
plt.savefig(f"only-corrected_spectrum.png")  # Save plot
plt.show()
```

Figure S14: Python code leading to the corrected diffuse reflectance spectrum generated by weighted interpolation of spectra measured under 350 nm and 420 nm lamp change. This correction improves spectral continuity across regions where the lamp changes. Only data from 200 to 500 nm are shown. The software can also be used for absorbance data.

Example of Diffuse Reflectance Spectra Before and After Correction

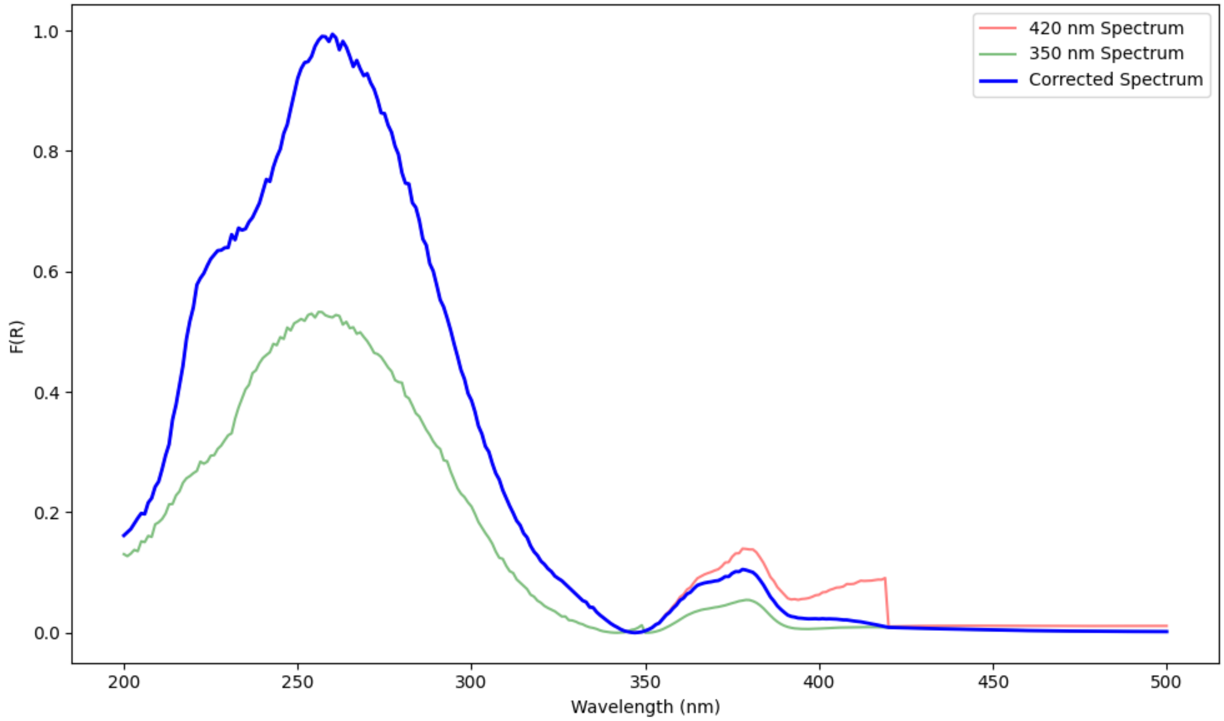

Figure S15: Diffuse reflectance spectra of TBADT@Alumina measured under lamp change at 350 nm (green) and 420 nm (red), along with the corrected spectrum (blue) generated by weighted averaging across the transition region (350–420 nm). Note that below 350 nm the blue and orange spectra overlap exactly. Above 420 the same occurs between blue and green spectra.

Diffuse Reflectance Spectra of Catalysts

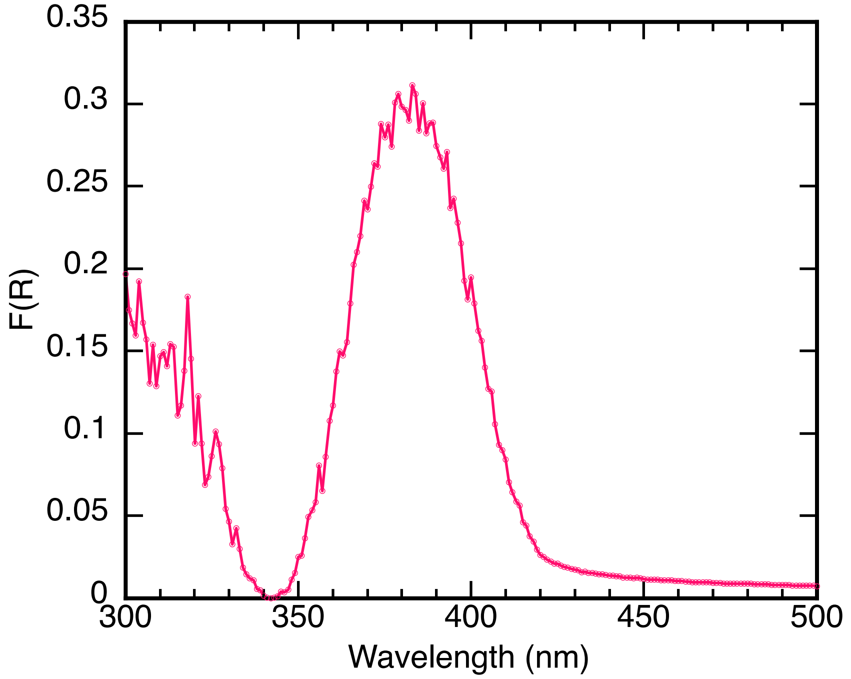

Figure S16: Diffuse Reflectance spectrum of TBADT@TiO_2, recorded using pristine TiO_2 as a reference.

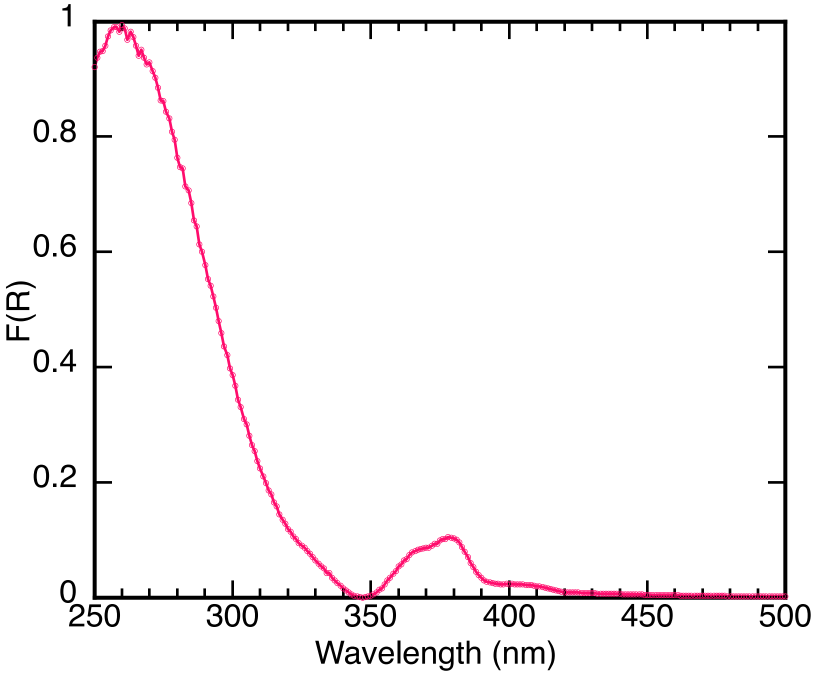

Figure S17: Diffuse Reflectance spectrum of TBADT@Alumina recorded using Spectralon as a reference.

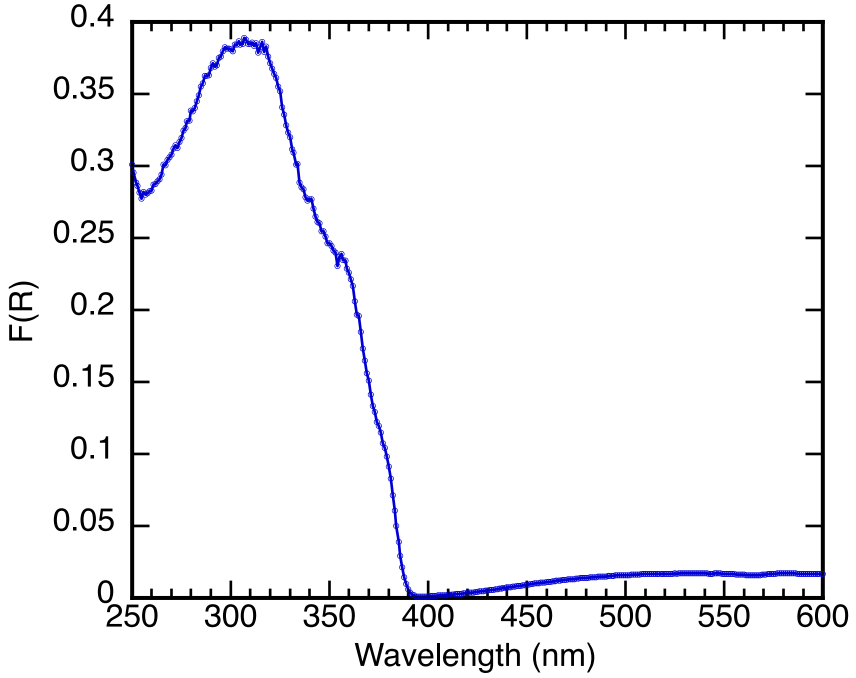

Figure S18: Diffuse Reflectance spectrum of Alumina (No Catalyst, Catalyst Preparation Procedure Repeated), recorded using Spectralon as a reference.

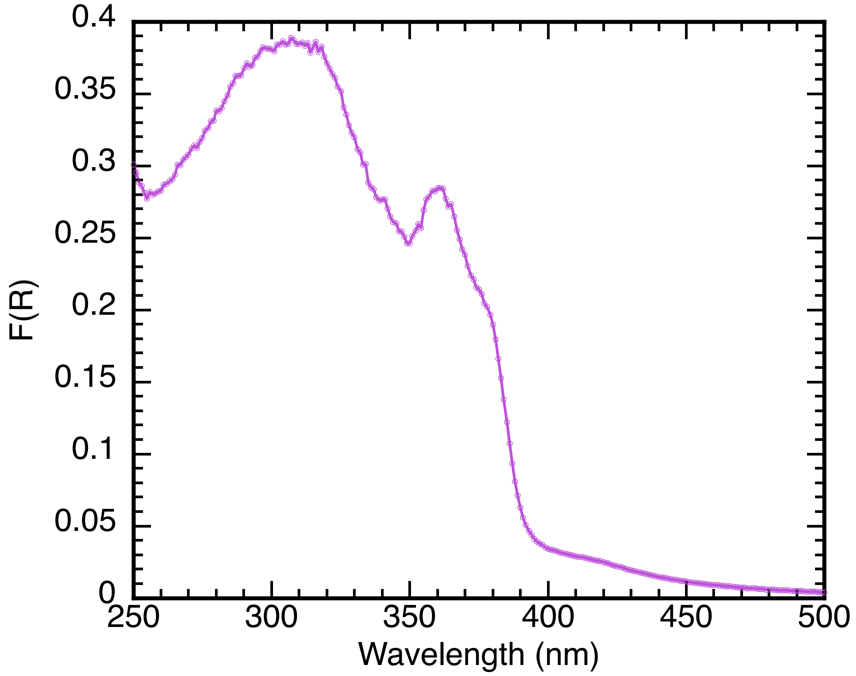

Figure S19: Diffuse Reflectance spectrum of Silica (No Catalyst, Catalyst Preparation Procedure Repeated), recorded using Spectralon as a reference.

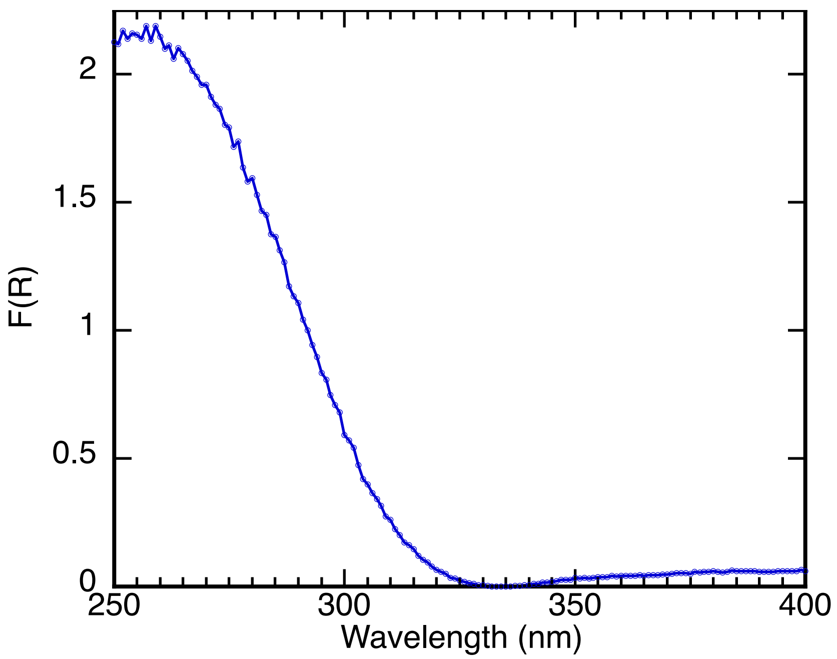

Figure S20 Diffuse Reflectance spectrum of TBADT@Silica-APTES, recorded using Spectralon as a reference. A lamp change at 420 nm can cause a deviation in the spectrum; for this reason, the scale is limited to <420 nm. Because of limited availability of material the correction used in other spectra could not be implemented.

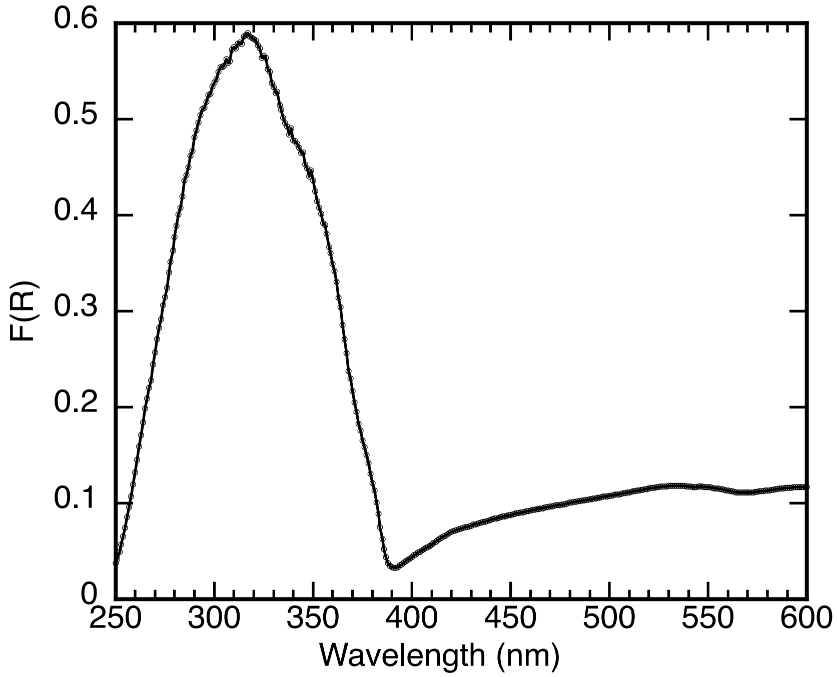

Figure S21: Diffuse Reflectance spectrum of NADT@Silica-APTES, recorded using Spectralon as a reference.

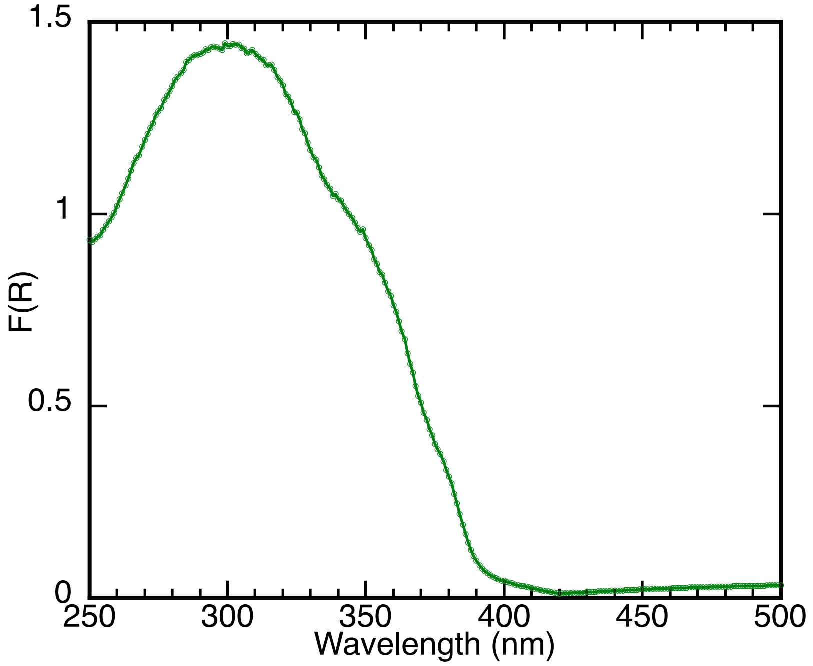

Figure S22: Diffuse Reflectance spectrum of NaDT@Silica, recorded using Spectralon as a reference.

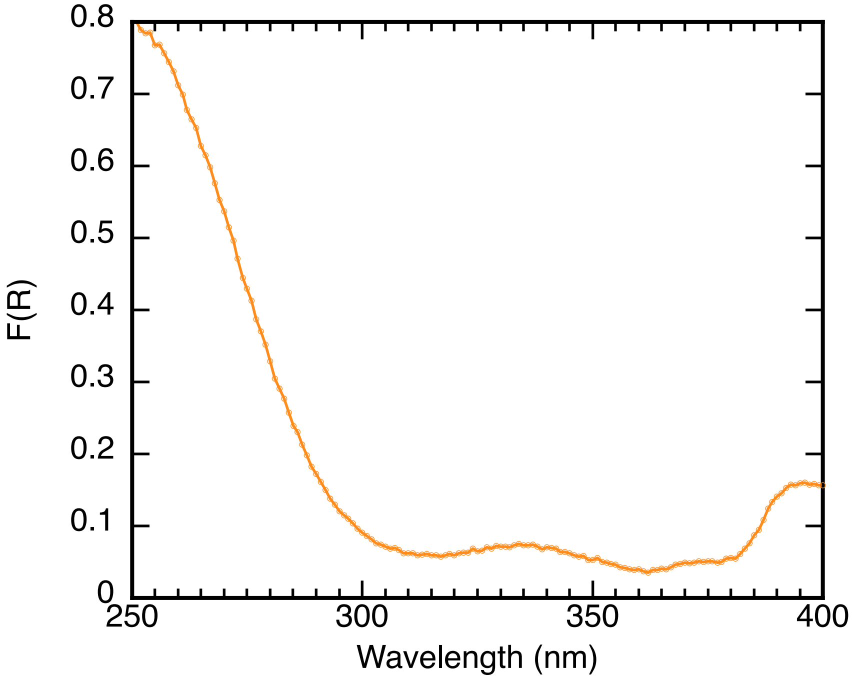

Figure S23: Diffuse Reflectance spectrum of TBADT@Glass Wool, recorded using Spectralon as a reference. A lamp change at 420 nm can cause a deviation in the spectrum; for this reason, the scale is limited to <420 nm. Because of limited availability of material the correction used in other spectra could not be implemented.

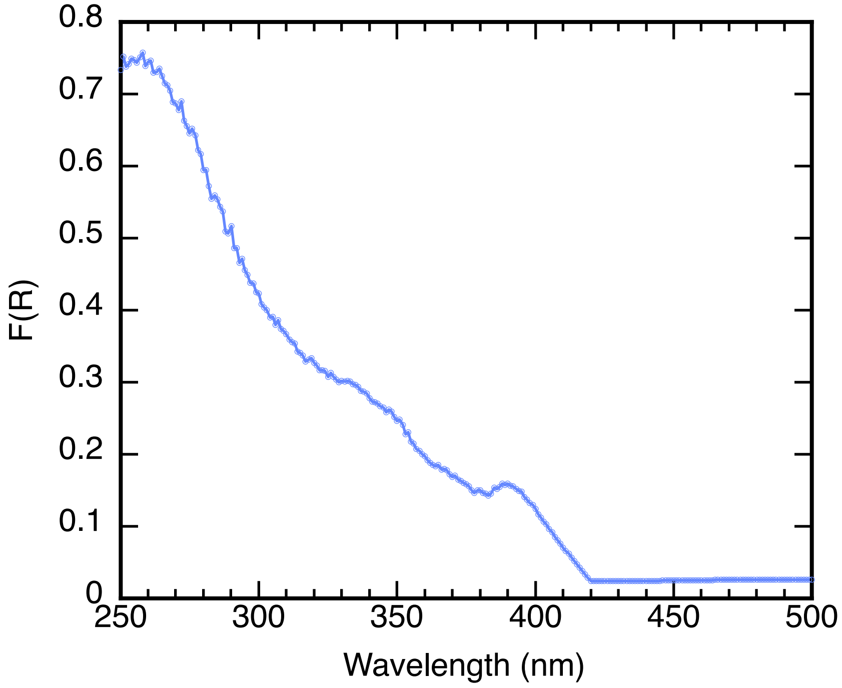

Figure S24: Diffuse Reflectance spectrum of TBADT@Glass Wool-APTES, recorded using Spectralon as a reference.

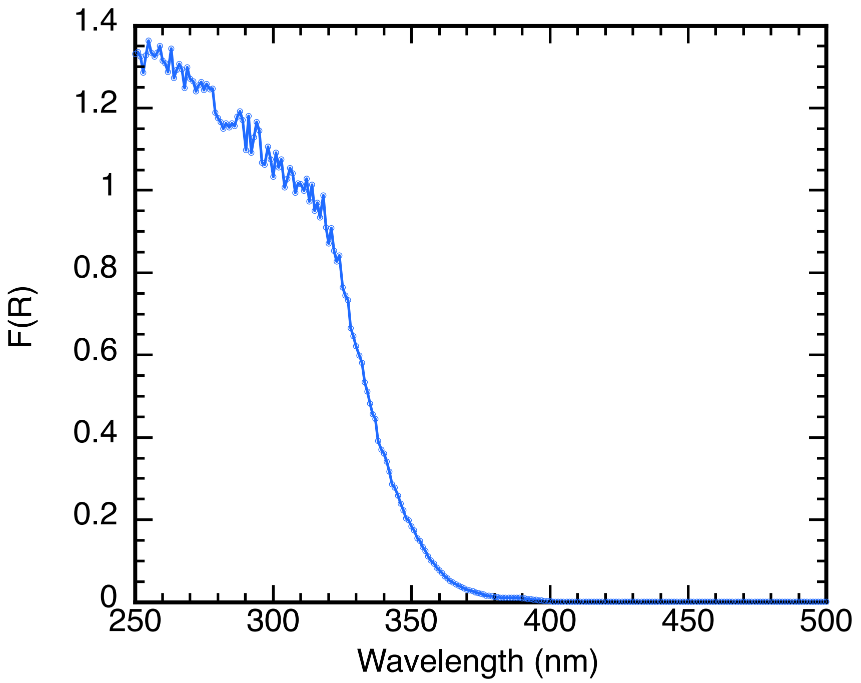

Figure S25: Diffuse Reflectance spectrum of TiO_2 (No Catalyst, Catalyst Preparation Procedure Repeated), recorded using Spectralon as a reference.

ICP Results of Catalyst following Acid Digestion Preparation

Calculated metal concentrations (ppm) in various catalyst samples as determined by ICP analysis. Samples were prepared using a wet acid digestion method with concentrated HNO_3 and HCl (aqua regia-assisted) prior to dilution and measurement. Unfortunately, results for DT@support samples gave some reproducibility problems, perhaps because the robust DT samples failed to dissolve quatitatively. and thus, to avoid confusion, we only provide general observations

- For TBADT@support samples, typically tungsten content was between 5 and 6% by weight, which is about 60% of the nominal loading based on the sample preparation in which 1 g of the DT salt was added to 5 g of support
- Loading for NaDT was only about one third of that achieved with TBADT

XRD Results of Catalysts

The X-ray diffraction (XRD) patterns of TBADT, NaDT and their heterogeneous catalysts were collected using a powder X-ray diffractometer (Bruker D8 Endeavor) with a diffracted beam monochromator Cu Kα source (40 kV-40 mA). Patterns were recorded from 2θ = 5 to 90°, in steps of 0.02°.

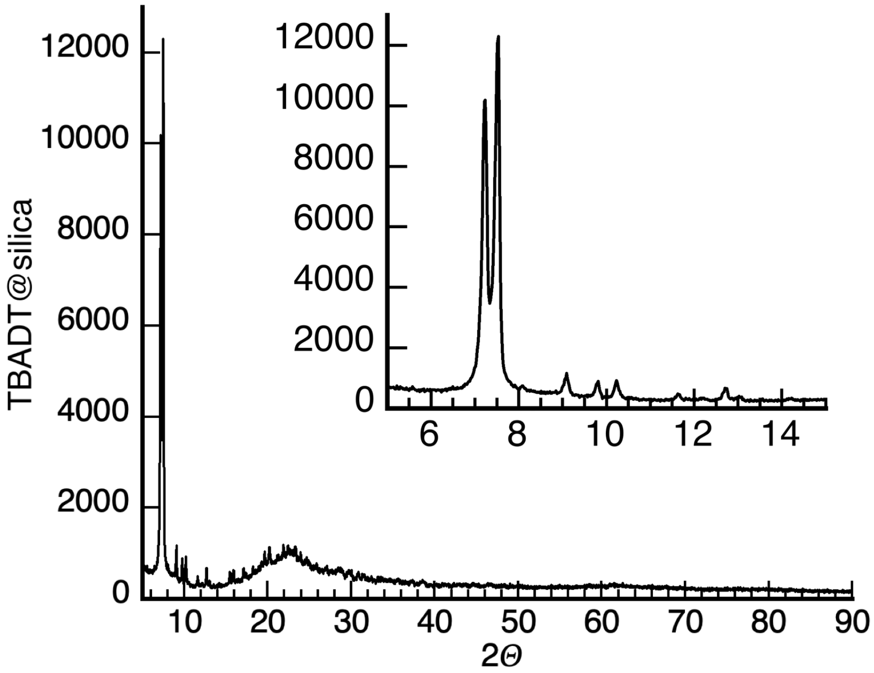

Figure S26: XRD spectrum of TBADT@silica powder, fresh before usage

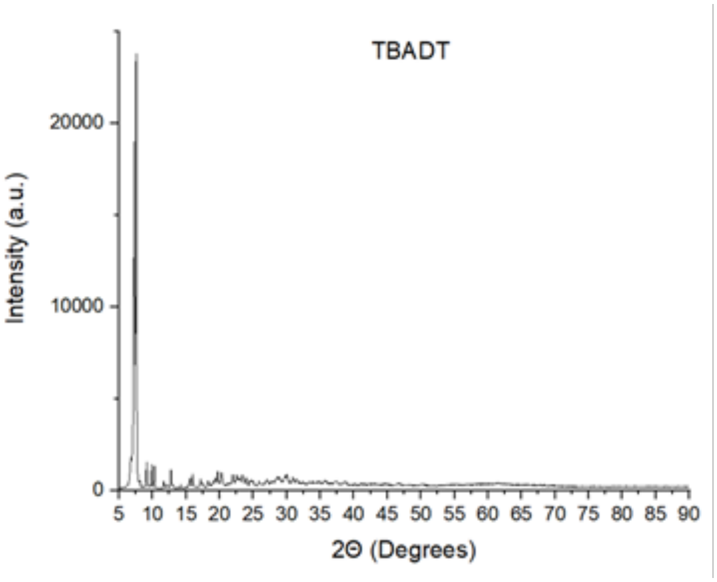

Figure S27: XRD spectrum of TBADT powder, no support

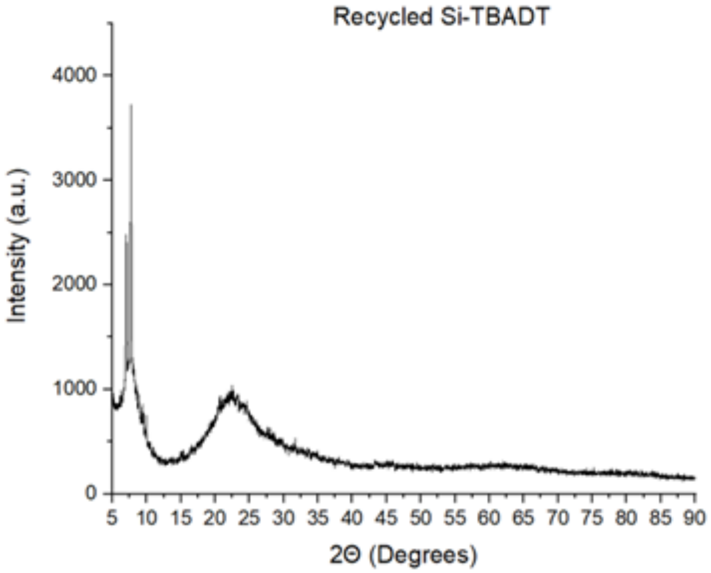

Figure S28: XRD spectrum of TBADT@silica powder, after one cycle

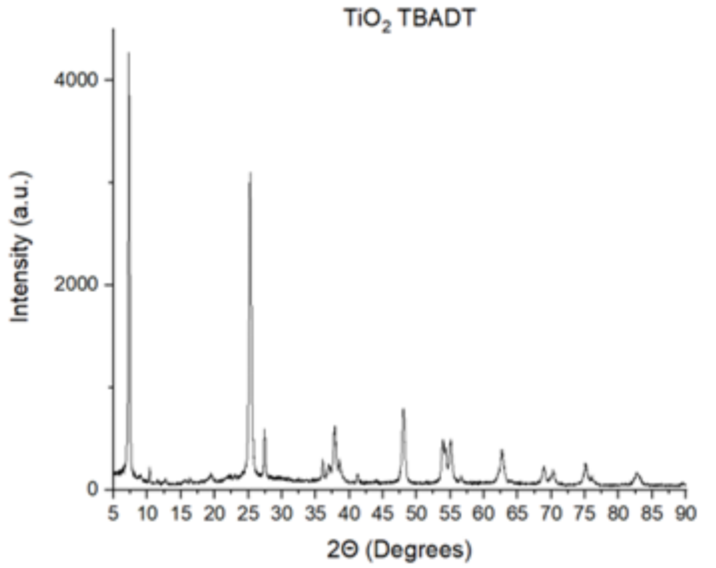

Figure S29: XRD spectrum of TBADT@TiO_2 powder, fresh before usage

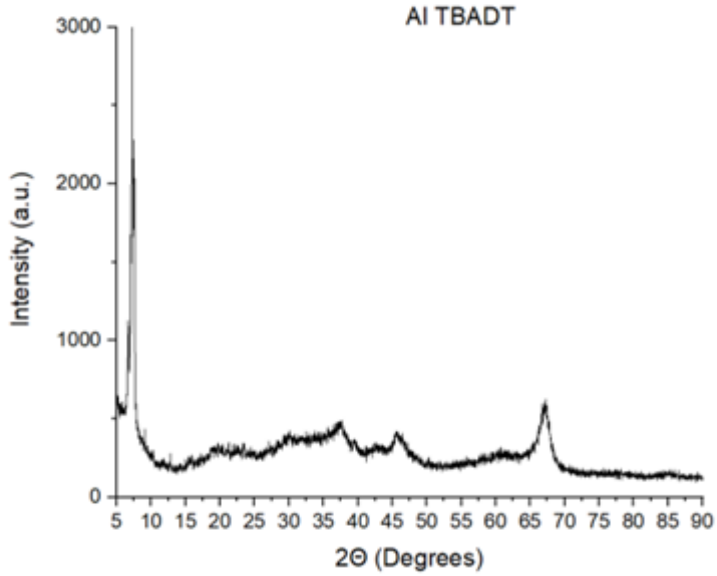

Figure S30: XRD spectrum of TBADT@alumina powder, fresh before usage

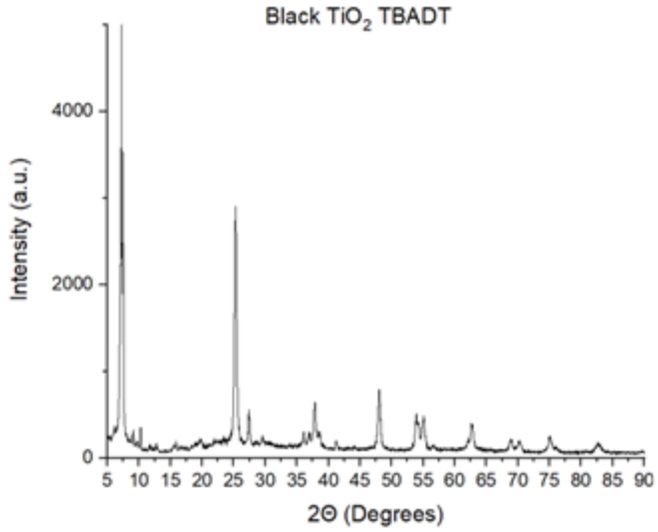

Figure S31: XRD spectrum of TBADT@black TiO_2 powder, fresh before usage

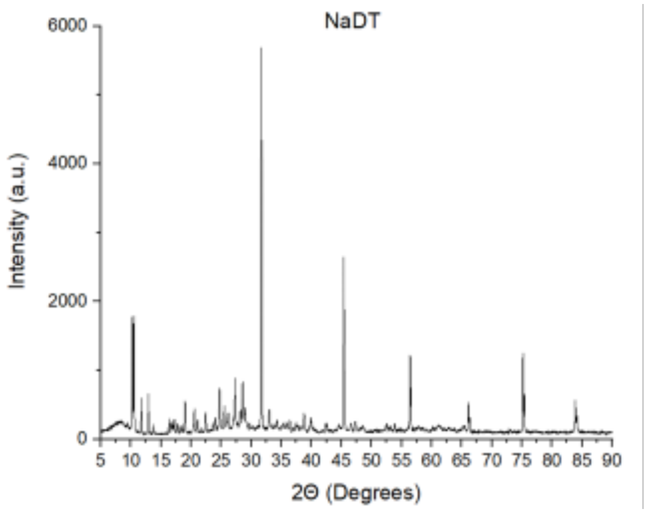

Figure S32: XRD spectrum of NaDT powder, no support

Table S1: Crystallite size calculations for TBADT, SiTBADT and recycled SiTBADT, all using the Scherrer equation

XRD conditions: Each sample was analyzed by powder XRD. TBADT's characteristic peaks at 7.2° and 7.6° are shown in the table below.

Sample‡	FWHI (°)	FWHI (rad)	2Θ (°)	Crystallite size (nm)
TBADT	0.1551	0.002706	7.255	51.4
TBADT	0.1558	0.002719	7.554	51.2
TBADT@silica	0.1352	0.002359	7.235	59.0
TBADT@silica	0.1340	0.002339	7.534	59.5
TBADT@silica R1	0.2030	0.003542	7.056	39.3
TBADT@silica R1	0.1943	0.003392	7.734	39.1

‡ The label R1 indicates that the sample was recycled. When no 'R' is shown, the sample is fresh

Tables showing the data on which various graphs are based

Table S2: Yields (%) obtained under UVA irradiation for 1-phenylethanol samples

Standard conditions: Each sample was irradiated in a fused quartz crimp vial and flushed with oxygen. The 10 mL vial contained 0.14 g of supported DT, 61 μL of 1-phenylethanol (0.1 M), 39 μL of internal tert-butyl benzene standard (0.05 M), and 5 mL of chloroform.

Support	DT type	Light	Treatment‡	0 h	2 h	3 h	4 h	24 h	46 h
Alumina	TBADT	UVA	None	2.9			4.76	20.63	
Alumina	TBADT	UVA	None - R2	0		0.98		7.27	
Alumina	TBADT	UVA	None- R1	0.29	0.96	2.9		11.09	
GW	None	UVA	APTES	2.06				10.43	
GW	TBADT	UVA	APTES	2.06[c]			3.88	23.01	
GW	TBADT	UVA	APTES	0.77		1.89		13.9	
GW	TBADT	UVA	APTES-R1	0.1		4.76		5.45	
GW	TBADT	UVA	None	0		0.95		16.67	
GW	TBADT	UVA	None-R1	0.48			1.84	12.15	
GW	TBADT	UVA	None-R2	1.46			1.63	2.53	
None	None	UVA	None	0.59[d]	0.65		0.85	3.65	
None	None	UVA	None	2.06			4.34	13.03	
None	None	UVA	None	0		0.61		4.46	
None	None	White	None	0		5.63		8.45	
Silica	NaDT	UVA	APTES	1.96		2.92		7.41	
Silica	NaDT	UVA	APTES	0.46		0.46		4.76	
Silica	NaDT	UVA	None	0		0.43		5.72	
Silica	None	UVA	None	0.58[d]	0.67		0.90	0.90	
Silica	TBADT	UVA	APTES	1		0		2.91	
Silica	TBADT	UVA	APTES	2.92		1.92		3.85	
Silica	TBADT	UVA	None	0.66[d]	2.33		3.56	12.1	

Support	DT type	Light	Treatment‡	0 h	2 h	3 h	4 h	24 h	46h
Silica	TBADT	UVA	None	1[e]	2.8		3.8	10	
Silica	TBADT	UVA	None	0.5[f]		2.2		8.5	
Silica	TBADT	UVA	None	4.8		9.9		54.5	
Silica	TBADT	UVA	None	0.5	1.87			49	65
Silica	TBADT	UVA	None	0.49	3.21		7.8	48.1	
Silica	TBADT	UVA	None - R3	0.5		1.19		5	
Silica	TBADT	UVA	None - R2	0.5		12.11		35	
Silica	TBADT	UVA	None- R1	0	0.93	1.64		39.8	
TiO_2	TBADT	UVA	None	1.63		6.43		27.3	
TiO_2	TBADT	UVA	None-R1	0.91[a]		1.31		9.64	
TiO_2	TBADT	UVA	None-R2	1.38[b]		1.51		6.1	
Black-TiO_2	TBADT	UVA	None	7.36		7.60		7.57	
Black-TiO_2	None	UVA	None	6.59		8.21		5.24	
Black-TiO_2	None	White	None	0		4.51		5.23	
Black-TiO_2	TBADT	White	None	0		10.9		6.34	

‡ The labels R1 and R2 indicate that the sample was recycled. When no 'R' is shown, the sample is fresh

[a] Only 0.10 g of catalyst recovered for R1 test

[b] Only 0.06 g of catalyst recovered for R2 test

[c] Only 0.06 g of catalyst were available for this test

[d] Liquid sample was 7 mL instead of the standard 5 mL

[e] 5 mL chloroform Silica-TBADT solution was rotated overnight (hot dog cooker); Catalyst was removed and supernatant was used for 1-phenylethanol oxidation

[f] 0.14 g of Silica-TBADT was suspended in 5 mL chloroform with 0.1 M 1-phenylethanol and 0.05 M tert-butylbenzene, irradiated under UVA light for 3 h, then the supernatant was transferred to a quartz vial for an additional 21 h of irradiation

Table S3: Yields (%) obtained under UVA and 280 nm irradiation for cyclohexanol samples

Note: Same conditions as in Table S1 except for the alcohol used.

**Support**	**DT type**	**Light**	**treatment**	**0 h**	**2 h**	**3 h**	**4 h**	**24 h**	**46**
None	None	UVA	none	0		0		1.5	
Silica	None	UVA	none	0		0		0.74	
Silica	TBADT	UVA	none	0.54	1.02			22.8	34.7
Silica	TBADT	UVA	none	0	1.79	2.47		22.2	
alumina	TBADT	UVA	none	0	0.72	1.15		6.4	
GW	TBADT	UVA	None	0		1.22		3.07	
TiO_2	TBADT	UVA	none	0		0.5		6.82	
None	None	280 nm	none	0			0.80	(20)[a]	
alumina	TBADT	280 nm	none	1.05			1.08	2.64	
Silica	TBADT	280 nm	none	1.67			2.96	(19.9)[a]	
GW	TBADT	280 nm	none	0.18			0.93	3.26	

[a] Numerous other products, yield is unreliable

BET Results for TBADT on Selected Supports

Detailed surface studies are not available at this time at the University of Ottawa and thi the measurements below are limited to key samples. Thet were obtained as a service from Carleton University (Ottawa, ON, Canada)

Table S4: Surface studies (BET)

Sample	Surface in m^2 per gram	Pore volume cm^3/g
TBADT@TiO_2	41.5	0.067
TBADT@black TiO_2	35.1	0.196
TBADT@alumina	97.3	0.056
TBADT@silica	102.5	0.502

Product Studies

Representative GC-MS chromatogram

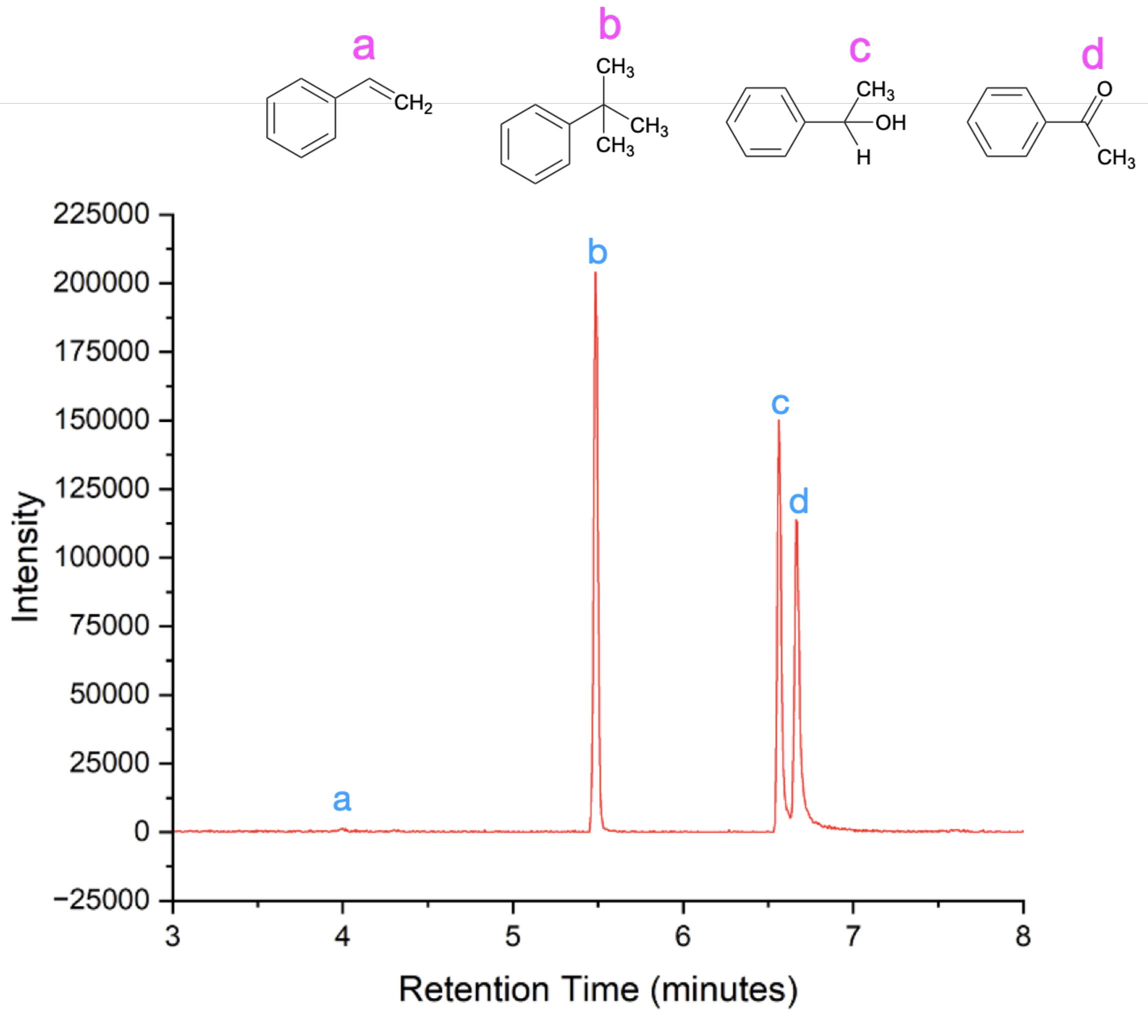

Figure S26: GC-MS of 0.14 g Silica-TBADT and 0.1 M 1-Phenylethanol in 5mL of $CHCl_3$ with 0.05 M tert-butylbenzene after 24 hours of UVA irradiation
